# LightIN: a versatile silicon-integrated photonic field programmable gate array with an intelligent configuration framework for next-generation AI clusters

**DOI:** 10.1038/s41377-026-02209-5

**Published:** 2026-03-11

**Authors:** Ying Zhu, Yifan Liu, Xinyu Yang, Kailai Liu, Xin Hua, Ming Luo, Jia Liu, Siyao Chang, Jie Yan, Shengxiang Zhang, Miao Wu, Zhicheng Wang, Hongguang Zhang, Dong Wang, Daigao Chen, Xi Xiao, Shaohua Yu

**Affiliations:** 1 National Information Optoelectronics Innovation Center, China Information and Communication Technologies Group Corporation, Youkeyuan Road 88, Wuhan, 430074 Hubei China; 2 State Key Laboratory of Optical Communication Technologies and Networks, China Information and Communication Technologies Group Corporation, Gaoxinsi Road 6, Wuhan, 430074 Hubei China; 3 Peng Cheng Laboratory, Shahexi Road 6001, Shenzhen, 518108 Guangdong China

**Keywords:** Integrated optics, Silicon photonics

## Abstract

Artificial Intelligence models pose serious challenges to intensive computing and high-bandwidth communication for conventional electronic circuit-based computing clusters. Silicon photonic technologies, due to their high speed, low latency, large bandwidth, and complementary metal-oxide-semiconductor compatibility, have been widely implemented for data transmission and actively explored as photonic neural networks in AI clusters. However, current silicon photonic integrated chips lack adaptability for multifunctional use and hardware-software systematic coordination, which is adverse to the development of photo-electronic AI clusters. Here, we develop a reconfigurable silicon photonic chip with 40 programmable unit cells integrating over 160 components, which, to the best of our knowledge, is the first to realize diverse functions for AI clusters with a chip, from computing acceleration and signal processing to network switching and secure encryption. Using a self-developed testing, compilation, and adjustment framework to the chip without in-chip monitoring photodetectors, we have demonstrated (1) 4 × 4 bi-direction unitary and 3 × 3 uni-direction non-unitary matrix multiplications, achieving a speed of over 1.92 TOPS with 6.22-bit precision and energy efficiency of 1.875 pJ MAC^−1^, and neural networks for image recognition with a latency of 260 ps; (2) micro-ring modulator wavelength locking in the 5 to 32 Gb s^−1^ transmission systems; (3) 4 × 4 photonic channel switching with low to –44 dB inter-channel crosstalk; (4) silicon photonic physical unclonable functions. This optoelectronic processing system, incorporating the photonic chip and its software stack, paves the way for both advanced photonic system-on-chip design and the construction of photo-electronic AI clusters.

## Abstract (web summary)

We demonstrate a programmable silicon photonic chip with an intelligent configuration framework, enabling on-chip computing, signal processing, switching, and encryption.

## Graphical abstract

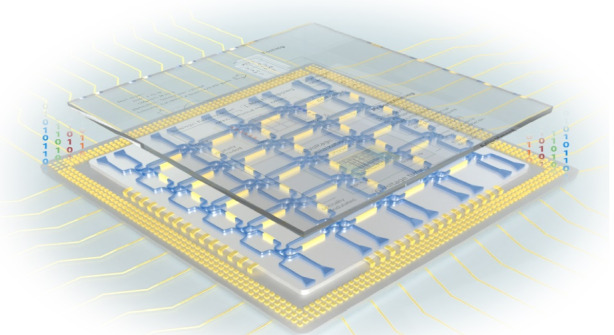

## Introduction

Artificial intelligence (AI) models demonstrate human-competitive performance in diverse fields, including natural language processing (NLP), healthcare, finance, education, autonomous driving, scientific research, creative industries, and more^1–5^. These intelligent capabilities are underpinned by large-scale computational resources processing vast amounts of data, often in petabytes to exabytes of training data and model parameters^6,7^. To meet these computational demands, current AI computing centers have evolved from clusters of thousands of Graphics Processing Units (GPUs) to large-scale systems comprising hundreds of thousands of accelerators^8^. However, conventional electronic-based computing hardware faces the Walls of Power^9^, Memory^10^, Interconnects^11^, and Speed^12^ due to the slowdown of Moore’s Law^13^, the von-Neumann architecture bottleneck^10,14^, and the increasing scaling of AI clusters. Consequently, these challenges necessitate the exploration of novel computing architectures and hardware solutions.

Silicon photonics has emerged as a promising solution to these challenges. It demonstrates superior performance in speed, energy efficiency, and latency, along with large bandwidth and complementary metal-oxide-semiconductor (CMOS) compatibility. Silicon photonic systems-on-chip (SoC) have achieved superior performance in various applications, including communication^15–17^, switching^18^, and computing^19–22^, all of which are critical requirements in AI clusters. Silicon photonic transceivers have become the mainstream solutions in intra- and inter-datacenter interconnects^23^. For shorter distances, optical input/output (I/O) achieves 4 Tb s^−1^ signal transfer with only 5 ns latency and 5 pJ bit^−1^ energy consumption, demonstrating 10× better performance in both speed and energy efficiency compared to electrical I/O^24^. In the field of AI computing, photonic computing has shown remarkable progress^25–27^. For instance, the large-scale photonic chiplet Taichi achieves 160 TOPS W^−1^ energy efficiency for AI acceleration^28^. A scalable photonic integrated circuit, which integrates multiple coherent optical processing units for linear and nonlinear functions on a single chip, demonstrates neural network computation with a latency of 410 ps^29^.

While these application-specific photonic integrated circuits (ASPICs) demonstrate impressive performance, their inherent functional limitations make it difficult to adapt to diverse application requirements. Furthermore, each ASPIC requires custom software development for configuration, lacking a universal hardware-software co-design framework. Besides, ASPIC development typically involves multiple design-fabrication-packaging-test iterations, with each iteration taking several months and incurring substantial development costs^30,31^. To overcome these limitations, researchers have proposed photonic field programmable gate arrays (P-FPGAs) or general-purpose processors, which are reconfigurable to realize diverse functions across different application fields, drawing inspiration from field programmable gate arrays (FPGAs) and central processing units (CPUs) in the electronic domain^32^. They promise to combine high performance (low cost, compactness, and energy efficiency) and rapid and economical functional verification and upgrade^31^. Current implementations include two significant architectures: forward-only and recirculating^31^. The forward-only architectures primarily employ Mach-Zehnder Interferometer (MZI)-based triangular mesh^33^ and rectangular mesh^34,35^. They enable unitary transformation from multiport inputs to outputs, supporting applications like quantum information processing^36^, neuromorphic computing^37^, mode conversion^38^, and signal processing^39^. However, they lack loop routing for infinite impulse response (IIR) filters and delay difference adaptation commonly used in signal processing and control systems. The recirculating waveguide meshes, with triangular, square, or hexagonal forms, achieve both IIR and finite impulse response (FIR) filters by programming to establish loop paths^40^. A hexagonal mesh comprising 72 programmable unit cells (PUCs) has successfully implemented key functions required in 5 G/6 G wireless systems, such as photonic and RF-photonic filtering, millimeter-wave generation, beamforming, frequency measurement, and optoelectronic oscillators^41^. A 9-cell square mesh demonstrates Hilbert transformation, temporal integration, routing, and matrix multiplications^42^. However, the configuration method for large-scale integrated circuits and their capability to support broader AI applications still need to be explored.

Here, we demonstrate a versatile silicon-integrated photonic field programmable gate array (P-FPGA) based on a 4 × 4 square recirculating mesh with 40 PUCs, one of the largest implementations to date. To achieve efficient and reliable programming and control, we develop a testing, compilation, and adjustment (TCA) framework. By incorporating the programmable photonic chip and the intelligent configuration framework with an electronic control module, we establish a comprehensive prototype processing system with **Light I**n the **N**etwork-on-Chip (LightIN) to realize diverse functions for AI clusters from computing acceleration and signal processing to channel switching and secure encryption. The processing system, LightIN, has completed 4×4 bidirectional unitary and 3 × 3 non-unitary matrix multiplications, achieving 1.92 TOPS at an energy efficiency of 1.875 pJ MAC^−1^ with over 6.22-bit computing resolution. We implement it as a neural network to perform an image-recognition task with the TCA framework, completing one image recognition in 260 ps with competitive accuracy compared to the electronic counterpart. For signal processing, it enables automatic wavelength locking of micro-ring modulators (MRMs) under 5 to 32 Gb s^−1^ non-return-to-zero (NRZ) modulation. The system also achieves 4 × 4-channel switching with –40 dB crosstalk over the 2.5 THz range, competitive with their counterparts. For secure encryption, we realize photonic physical unclonable functions (PPUFs) on the LightIN, achieving an intra-die Hamming distance of 1.7% and an inter-die Hamming distance of 50.15%. The TCA framework and the P-FPGA establish the foundation for developing large-scale photonic integrated circuits and photo-electronic AI clusters.

## Results

### Prototype system: architecture and configuration

**Fig. 1:**
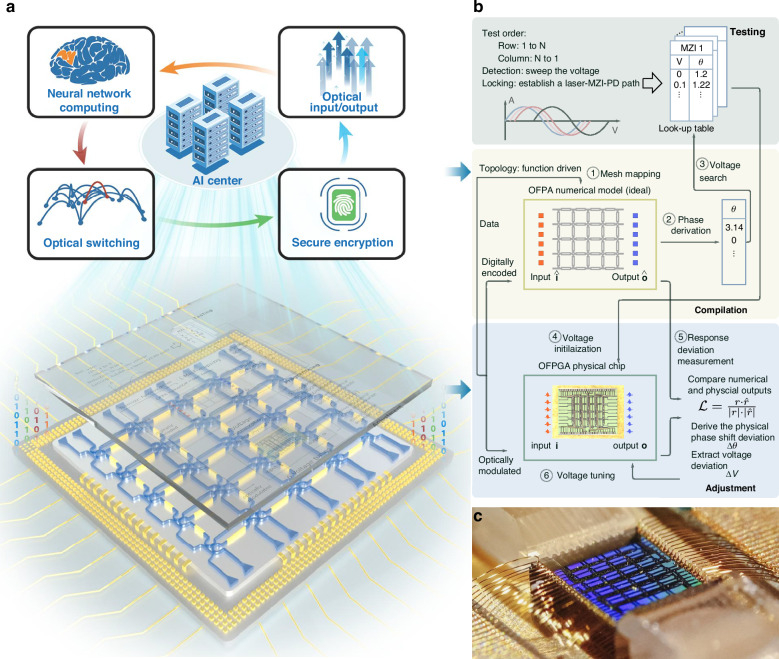
The silicon photonic processing system, LightIN, and its applications in AI clusters. **a** The square recirculating mesh P-FPGA conceptual diagram and its supporting functions in AI clusters. **b** The testing, compilation, and adjustment (TCA) framework. **c** Photograph of the packaged silicon-integrated photonic chip

The prototype system LightIN consists of a silicon-integrated P-FPGA and an electronic control module at the hardware level, complemented by a TCA framework at the software level, as shown in Fig. 1. The photonic chip, fabricated using silicon-on-insulator (SOI) technology, features 40 PUCs arranged in a flat 4 × 4 square mesh topology (Fig. 1). Twenty optical ports are equally distributed at the two opposite edges of the chip for signal input/output via two fiber arrays. Each PUC is an MZI with a thermo-optic phase shifter ${\rm{\theta }}$ on one arm, whose transformation matrix is

1 $${T}_{{PUC}}=j{e}^{j\frac{\theta }{2}}\left[\begin{array}{cc}\sin \frac{\theta }{2} & \cos \frac{\theta }{2}\\ \cos \frac{\theta }{2} & -\sin \frac{\theta }{2}\end{array}\right]$$

By applying voltages to ${\rm{\theta }}\in$PUCs, the PUC and the PUC-based square mesh transformation matrices can be configured to process versatile functions. The voltage configuration is implemented by the electronic control module. It interfaces with the silicon photonic chip via a Printed Circuit Board (PCB), to which the PUCs are electronically connected through wire bonding. Details of the photonic chip are provided in Section Materials and Methods.

To systematically program the photonic chip, we designed and implemented a three-phase intelligent configuration framework, the TCA framework (Fig. 1b), detailed as follows:

#### Testing: MZI characterization

We propose a testing protocol to establish the relationship between the MZI states and applied voltages, which could vary among different dies due to fabrication variations and hinder function compilation. The testing protocol progressively characterizes all MZIs in the mesh through alternating detection and locking. The characterization sequence follows a specific pattern: progressing row by row from the first to the last, and within each row, traversing from the rightmost to the leftmost column. For each MZI:

(1) Detection for MZI states: sweep the control voltage, measure output intensities, derive programmed phase shifts via Eq. (1), and build the voltage-phase Look-Up Table (LUT). The cross and bar states are identified from intensity extrema. An example of the characterization result for an MZI is presented in the supplementary information note 1.

(2) Locking MZI states for path generation: program pre-tested MZIs to cross/bar states according to their LUTs, establishing a unidirectional optical path from the tested MZI to the output. This path, which is ensured by the characterization sequence, provides intensity measurements directly correlated with the tested MZI’s phase-voltage response. (While the untested MZI states are indefinite due to the manufacturing variations, paths generally exist from the input to the tested MZI.)

Repeat the Detection step for all MZIs to complete the characterization process. This hierarchical approach enables reliable voltage initialization for subsequent function compilation. The process time is proportional to the number of MZIs and the voltage sweeping step size. Moreover, the characterization process is a one-time initialization process before its deployment for various functions, and the time cost is negligible compared to the chip’s operational lifetime.

#### Compilation: programming voltage initialization

The compilation phase determines the MZI phase values and the corresponding initialization voltages required by the tasks.

**Fig. 2:**
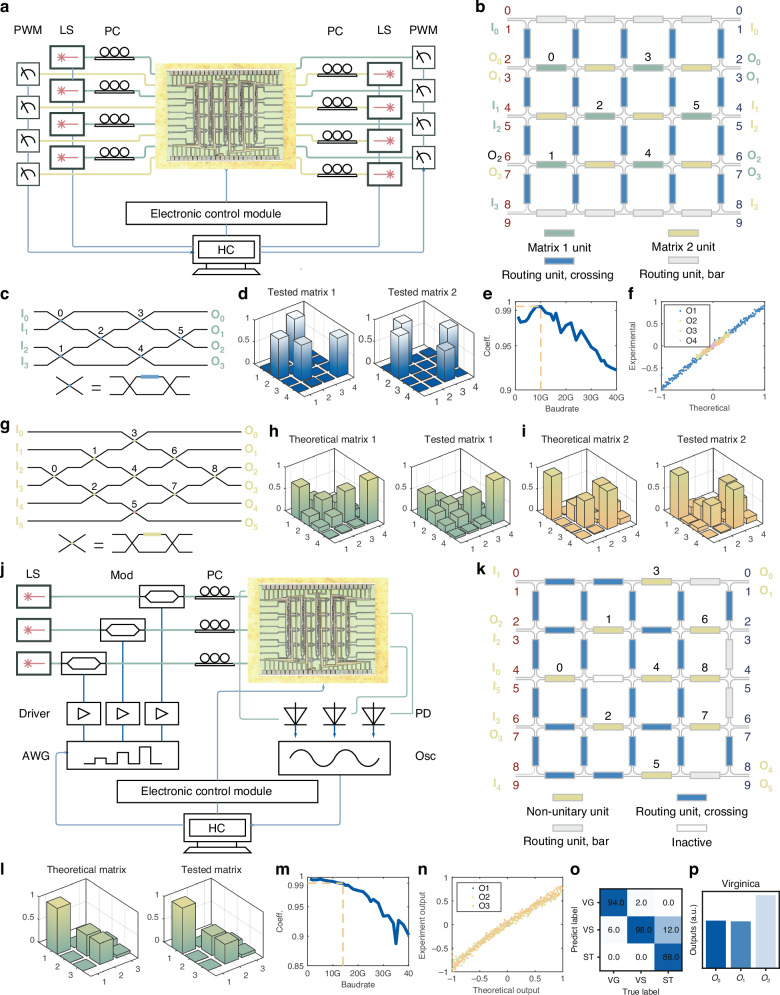
Experimental system and results for optical computing implemented on LightIN. **a** Experimental system for bidirectional unitary matrix expression. **b** Programming topology of LightIN for unitary matrix multiplication. **c** MZI-based rectangular mesh for unitary matrix. **d** Unitary matrix expressions of matrices $[[0,0,1,0],[1,0,0,0],[0,1,0,0],[0,0,0,1]]$ and $[[0,1,0,0],[1,0,0,0],[0,0,0,1],[0,0,1,0]]$ on LightIN. **e** Correlation coefficient variation with signal baud rate for unitary matrix multiplications. **f** Comparison between experimental and theoretical outputs of unitary matrix multiplication under 10 Gbaud rate. **g** Diamond mesh for the non-unitary matrix. **h** Comparison between theoretical and experimental matrices of a randomly generated unitary matrix. **i** Comparison between theoretical and experimental matrices of another randomly generated unitary matrix. **j** Experimental system for non-unitary matrix multiplication. **k** Programming topology of LightIN for non-unitary matrix multiplication. **l** Comparison between theoretical and experimental matrices of a randomly generated non-unitary matrix. **m** Correlation coefficient variation with signal baud rate for non-unitary matrix multiplications. **n** Comparison between experimental and theoretical outputs of non-unitary matrix multiplication under 10 Gbaud rate. **o** Confusion matrix of Iris dataset inference. **p** Example of output intensity distribution for class “Virginica”. PWM power meter, LS laser source, PC polarization controller, Mod modulator, AWG arbitrary waveform generator, PD photodetector, Osc oscilloscope, HC host computer

(1) Topology selection: deploy the predetermined MZI mesh according to the tasks, determining the routing MZIs and functional MZIs. For example, unitary matrix implementations in MZI-based photonic chips conventionally utilize the rectangle mesh topology^34^, determining the vertical and edge horizontal MZIs and parts of the horizontal MZIs in the square mesh as the routing MZIs and functional MZIs, respectively, as in Fig. 2.

(2) Phase shifter calculation: calculate the phase values according to the predetermined MZI mesh topologies. For the unitary matrices, phase values of the routing MZIs are fixed to 0 or ${\rm{\pi }}$ for the bar or cross states, and the phase values of the functional MZIs are obtained by decomposing the target unitary matrix.

(3) Initialization voltage determination: determine the initial programming voltages by searching the voltage-phase LUTs based on the derived phase values.

Additional topologies and corresponding phase values for tasks in signal processing, network switching, and secure encryption are presented in the following sections.

#### Adjustment: numerical and physical adjoint tuning

The adjustment phase aims to mitigate multi-disturbances: thermal crosstalk during programming and environmental noises. We establish an adjoint tuning method by constructing a digital-twin square mesh numerical model and comparing the simulated output-to-input responses $\hat{r}$ and measured ones $r$ from the physical chip by ${\mathcal{L}}=r\cdot \hat{r}/(|r|\cdot |\hat{r}|)$. Afterward, the programming voltages are tuned online according to their gradients with respect to ${\mathcal{L}}$, which are obtained via voltage adjustments and observations of ${\mathcal{L}}$ changes. The adjustment is significant for the phase-sensitive tasks. It should be noted that the adjoint tuning is applied only once during the application deployment and the TCA energy consumption is not accounted for in the operations of the following experimental demonstrations.

The TCA framework provides a systematic intelligent configuration approach for the following experiments and practical applications of the LightIN to AI clusters, ranging from computing acceleration and signal processing to optical switching and secure encryption functionalities.

### Computing acceleration in AI clusters

AI computing plays a crucial role in modern AI clusters. For high-speed and energy-efficient computing, photonic chips emerge as promising hardware solutions due to their high bandwidth and passive characteristics^37,43,44^. In this section, we utilize the LightIN to realize bidirectional unitary matrix multiplication, non-unitary matrix multiplication, and neural network computing.

#### Bidirectional unitary matrix multiplication

Figure 2a presents the experimental setup for the bidirectional unitary matrix expression on LightIN. Figure 2b demonstrates its programming topology based on the universal multiport interferometer structure (Fig. 2c). The photonic chip comprises four categories of PUCs: matrix 1 units (orange), matrix 2 units (green), and routing units (blue and gray). This interleaved programming and routing multiplexing improves the footprint efficiency by a factor of 1.95 compared to the hexagonal topology (detailed in the supplementary information note 2).

We first simultaneously implement two 4 × 4 unitary matrices on the LightIN with the TCA framework, one of which is the transmission matrix of the programmable photonic chip from left to right and the other from right to left. We input two 4×4 identity matrices through input ports from both directions, with each column being sent sequentially. The corresponding outputs are representative of the two transmission matrices. Figure 2d presents the tested unitary matrix 1 and unitary matrix 2, showing a high fidelity with the target ones, $\left[\left[0,0,1,0\right],\left[1,0,0,0\right],\left[0,1,0,0\right],\left[0,0,0,1\right]\right]$ and $[[0,1,0,0],[1,0,0,0],[0,0,0,1],[0,0,1,0]]$. Additionally, two randomly generated unitary matrices are implemented on LightIN. Figure 2h, i illustrates the modulus of the elements in the unitary matrices, which align well with their theoretical values. The results demonstrate the capability of the LightIN architecture for bi-directional data processing.

Furthermore, we input 256 trials of different 4 × 1-column vectors to the P-FPGA. The vector values are encoded as the amplitudes of return-to-zero (RZ) pulses under different baud rates. Figure 2e shows the correlation coefficient between the theoretical and experimental computing results under different data baud rates. As the baud rate increases, the correlation coefficient decreases, suggesting that the computing result resolution decreases. It could not be caused by the MZI-mesh bandwidth but by the limited AWG sampling rate, which generates the modulation RZ pulses, and the wavelength sensitivity of the grating couplers for input/output of the silicon-integrated P-FPGA. When the baud rate is 10 GBaud, the normalized output intensities and theoretical values present a high correlation of 0.9955 with $\sigma =0.0269$, corresponding to 6.22 bit^14^, depicted in Fig. 2f. The computing speed achieves 1.92 TOPS, and the average energy efficiency for the photonic core is 1.875 pJ MAC^−1^. A comprehensive energy efficiency analysis of the P-FPGA core and system is provided in the supplementary information note 3.

#### Non-unitary matrix multiplication

To realize non-unitary matrix multiplications, we implement a diamond structure (Fig. 2g), which has the advantages of uniform layout and straightforward programming procedure^45,46^, rather than two MZI-based unitary meshes and a group of parallel MZIs, amplifiers, or attenuators. The mathematical derivation for implementing a non-unitary matrix using the diamond mesh is described in the supplementary information note 4, while the advantage analysis for the diamond structure is detailed in the supplementary information note 5.

While due to chip size limitations, a completely forward-only diamond mesh cannot be established on the chip, more than one available topology exists on the square mesh to realize a 3 × 3 non-unitary matrix with the diamond structure. We can fold the diamond structure with some column as the axis, and locate the MZIs after the column of the diamond structure to the topologically equivalent MZIs in the square mesh, as shown in Fig. 2k. This characteristic demonstrates that the P-PFGA not only has flexibility but also improves footprint efficiency and fault tolerance.

To validate the design, we implement a randomly generated 3 × 3 non-unitary matrix on LightIN. The modulus of the non-unitary matrix elements, as illustrated in Fig. 2l, demonstrates remarkable agreement with the theoretical calculations. Additionally, we conduct 256 trials with distinct 3 × 1 column vectors on the experimental setup as shown in Fig. 2j, the values of which are encoded as the amplitudes of the RZ pulses. The correlation coefficients between experimentally obtained results and theoretical outputs, observed under varying baud rates, are displayed in Fig. 2m. The baud rate can achieve a speed of 14 GBaud with a correlation coefficient greater than 0.99. When data are modulated at 10 Gbaud, the correlation coefficient achieves 0.9930 ($\sigma =0.0453$), the resulting output intensities exhibit an effective bit resolution of 5.47 bit, closely aligning with theoretical outputs as shown in Fig. 2n.

#### Neural network for image recognition

To demonstrate the computational capabilities of LightIN for neural network applications, we implement a one-layer neural network on our chip and evaluate it using the Iris dataset, which comprises 150 samples with 4 numeric features across 3 classes. We perform offline training of a unitary neural network structure that can be represented by the photonic chip, achieving an inference accuracy of 94.67%. After programming the trained phase shift values into the photonic chip, we obtain online inferences with an accuracy of 93.33%. Figure 2o presents the inference confusion matrix, along with an example (Fig. 2p) of the output intensity distribution corresponding to the Versicolor, Setosa, and Virginica. The on-chip signal latency is approximately 60 ps, with a maximum signal path length of 4.5 mm, assuming a group index of 4. Including a pulse width of 100 ps (a baud rate of 10 Gbaud), the total latency remains below 260 ps. These results demonstrate that the silicon-integrated programmable photonic chip can effectively implement AI computing with competitive inference accuracies compared to conventional electronic counterparts.

#### Signal processing for Optical I/O in AI clusters

With electrical I/O nearing the bandwidth-distance limitations, optical I/O based on MRMs has emerged as a compelling solution for achieving long-distance, high-bandwidth transmission within AI centers^47^. However, the performance of MRMs is highly sensitive to thermal fluctuations, resulting in the degradation of modulation depth and the increase of the bit error rate (BER) of transmission links. To achieve a high extinction ratio (ER) for modulated signals, we present, to the best of our knowledge, the first exploration of utilizing a programmable photonic chip in the control strategy, which processes optical symbols with low latency and high energy efficiency.

**Fig. 3:**
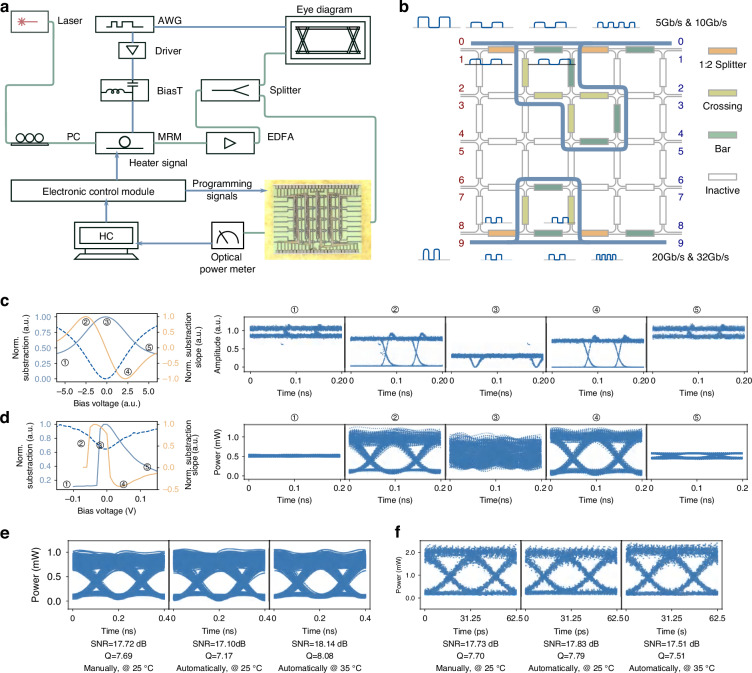
Experimental system and results of LightIN-based automatic wavelength locking for MRMs in optical I/O. **a** Experimental setup for LightIN-Based Wavelength Locking of MRMs. **b** Programming topology of LightIN as a photonic differentiator for MRM wavelength locking. **c** Normalized output optical power of the photonic differentiator and corresponding MRM eye diagrams under different heater voltages in 10 Gb s^−1^ NRZ modulation simulation. **d** Normalized output optical power of the photonic differentiator and corresponding MRM eye diagrams under different heater voltages in 10 Gb s^−1^ NRZ modulation experiment. **e** Eye diagrams of MRMs operated under 5 Gb s^−1^ NRZ modulation. **f** Eye diagrams of MRMs operated under 32 Gb s^−1^ NRZ modulation

The photonic circuit-based control strategy is developed based on the fact that a high ER indicates a significant amplitude difference between the pulse logic ‘1’ and ‘0’. To measure this amplitude difference, we configure the chip as a differentiator as in Fig. 3b. The input signals are divided by a splitting PUC (orange MZI) with ${\rm{\theta }}={\rm{\pi }}/2$ as in Eq. (1), generating two identical signals with half intensity. These signals travel through two distinct paths with different delays, which are constructed by different numbers of PUCs (green MZIs) employing different delays. It creates a time misalignment between the two signals at the combining PUC (orange MZI) with ${\rm{\theta }}=3{\rm{\pi }}/2$ as in Eq. (1). The combined signal represents the complex subtraction between the misaligned symbols at light speed. The subtracted light signal is converted into an electronic monitoring signal through photo-electronic conversion. This monitoring signal is proportional to the square of the complex amplitude difference between misaligned symbols. For example, when a 10 Gb s^−1^ pulse sequence of [0,1,0] experiences a 0.1 ns time misalignment between two paths, the combined pulse sequence becomes $[\Delta 0,\Delta 1,\Delta 1,\Delta 0]$, where $\Delta 1$ represents the amplitude difference between the pulse logic ‘1’ and ‘0’ and $\Delta 0$ represents the amplitude difference between two pulses with the same logic. A significant pulse amplitude difference, indicating high ER, leads to a high combined pulse sequence power, and vice versa. When the MRM bias approaches the optimal wavelength locking position (marked as ② in Fig. 3c), the combined sequence power exhibits a significant fluctuation due to rapid variations in ‘1’ and ‘0’ pulse intensities. Notably, at the micro-ring resonance wavelength, the modulated signals experience a ${\rm{\pi }}$-phase jump. When modulation signals are on the two sides of the resonance wavelength, the complex amplitude subtraction becomes equivalent to the absolute value addition of amplitudes. Consequently, as the MRM resonance wavelength approaches and deviates from the laser source, the electronic monitoring signal power grows and declines accordingly. At the critical points of growth and decline, the modulated signal achieves a high ER, as depicted in the simulation results (Fig. 3c). Therefore, we can utilize a micro-control circuit to read the electronic monitoring signal, track its variation, and generate the MRM heater-adjusting signal, increasing or maintaining a high ER. The feedback loop is from the MRM output, through the LightIN-based differentiator, and micro-control circuit to the MRM heater.

We establish an experimental system as in Fig. 3a. Since the symbol period varies with the baud rate, the delay difference in the photonic differentiator should be adjusted accordingly. As shown in Fig. 3b, the P-FPGA achieves this adaptation through programming. In the 10 Gb s^−1^ NRZ modulation system, by manually adjusting the heater voltage, the obtained eye diagrams, symbol subtraction and the subtraction slope, and MRM output power demonstrate good agreement with the simulation results as in Fig. 3c, d. This agreement verifies the principle correctness of the proposed strategy.

Furthermore, we implement the automatic control approach for MRMs with 5 Gb s^−1^ and 32 Gb s^−1^ NRZ modulations, respectively. First, they operate under the 25 °C environment controlled by a thermo-electronic cooler (TEC). The automatic control approach adjusts the MRM to its optimal working point, resulting in the eye diagrams as shown in Fig. 3e, f marked with “Automatically, @25 °C”. The measured Signal-to-Noise Ratios (SNRs) are 17.10 dB and 17.83 dB, and the Quality factors (Q factors) are 7.17 and 7.79 for 5 Gb s^−1^ and 32 Gb s^−1^, respectively. Subsequently, the TEC adjusts the working temperature to 35 °C. The control approach automatically searches and adjusts the heater voltage, producing the eye diagrams shown in Fig. 3e, f marked with “Automatically, @35 °C”. At this temperature, the SNRs are 18.14 dB and 17.51 dB, and the Q factors are 8.08 and 7.51 for 5 Gb s^−1^ and 32 Gb s^−1^, respectively. It has to be acknowledged that the y-axis values of the eye diagrams under different modulation baud rates are not comparable due to the use of Erbium-Doped Fiber Amplifiers (EDFAs) with varied amplification factors, which is only for better eye diagram visualization. The eye diagrams, SNRs, and Q factors under different modulation speeds and operating temperatures fit well with those obtained through manual adjustment (marked with “Manually, @25 °C“ in Fig. 3e, f), demonstrating that the LightIN-based automatic locking hardware can effectively align the MRM optimal working wavelength to the laser source. These results confirm that by configuring differentiators in the LightIN P-FPGAs, efficient wavelength locking can be achieved for MRMs, paving the way for stable optical I/O operations in large-scale AI clusters. A more comprehensive analysis of the advantages of the photonic differentiator-based approach is detailed in the supplementary information note 6.

### Optical switching in AI clusters

To accommodate the rapidly growing data amounts and transmissions in increasingly scaling AI clusters, silicon photonic integrated switching chips have emerged as a promising technology, offering advantages such as adaptive resource allocation, low latency, high bandwidth, high energy efficiency, and signal transparency. There are diverse MZI-based switching topologies, including the rearrangeable non-blocking N-stage planar and wide-sense non-blocking path-independent loss (PILOSS) structures^48^.

**Fig. 4:**
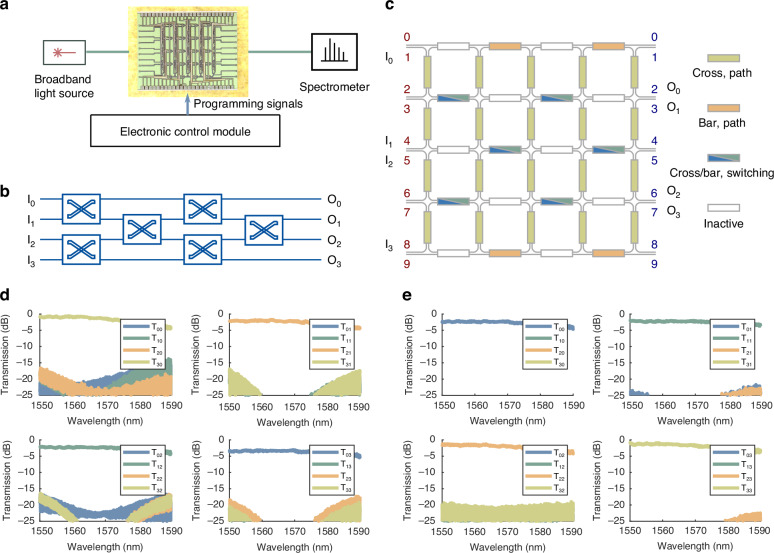
Experimental system and results for optical switching implemented on LightIN. **a** Experimental setup for measuring switching spectra on LightIN. **b** Schematic of the 4-stage planar switching structure. **c** All-cross/all-bar state programming topology of LightIN. **d** The normalized transmission spectra for the all-cross state, where ${T}_{{ij}}$ represents the transmission spectrum from input port $j$ to output port $i$. **e** The normalized transmission spectra for the all-bar state, where ${T}_{{ij}}$ represents the transmission spectrum from input port $j$ to output port $i$

Here, we demonstrate a 4-stage planar switching structure (Fig. 4b) on the LightIN, which eliminates optical crossovers and offers a rearrangeable non-blocking operation. To evaluate its performance, we measure the spectral characteristics of all optical paths using the experimental setup shown in Fig. 4a. The results demonstrate that the inter-port crosstalk ranges from –45 dB to below –20 dB at the central wavelength of 1560 nm. Across a bandwidth exceeding 20 nm, the crosstalk remains below –15 dB and –20 dB for the all-cross (Fig. 4b) and the all-bar (Fig. 4c) states, respectively.

Regarding the insertion loss, we have measured four optical paths under both states. In the all-bar state, the average insertion losses across over 40 nm are –2.76 dB, –2.47 dB, –2.23 dB, and –1.98 dB, while in the all-cross state, they are –1.85 dB, –2.64 dB, –2.62 dB, and –2.99 dB (excluding vertical coupler losses). The optical path from arbitrary input to arbitrary output includes a pair of vertical couplers and an identical number of MZIs, resulting in the uniform loss among different paths, as shown in Fig. 4c.

The LightIN-based switching performance can be further improved through the optimization of MZI designs and fabrication processes to achieve higher extinction ratios. The PILOSS structure, with the advantage of achieving uniformity loss across all paths, can also be implemented in larger-scale LightIN chips in future work, as detailed in supplementary information note 7. In summary, our experimental results confirm the feasibility of the LightIN for optical switching implementations, showing promise for high-efficiency data transmission applications for AI clusters.

### Secure encryption for AI clusters

The rapid development of AI computing and massive data transmission has increased information security requirements^49^. While traditional security systems storing the secret keys in nonvolatile memory (NVM) are vulnerable to external attacks or require complex circuits, PUFs, taking advantage of their inherent hardware randomness from the manufacturing process, can act as the “fingerprint” for the computing systems^50^. When an input challenge $C$ stimulates the PUF, it outputs the response $R=f(C)$, where $C$ and $R$ are multi-bit data and $f(\cdot )$ is determined by the PUF design structure and its manufacturing hardware.

**Fig. 5:**
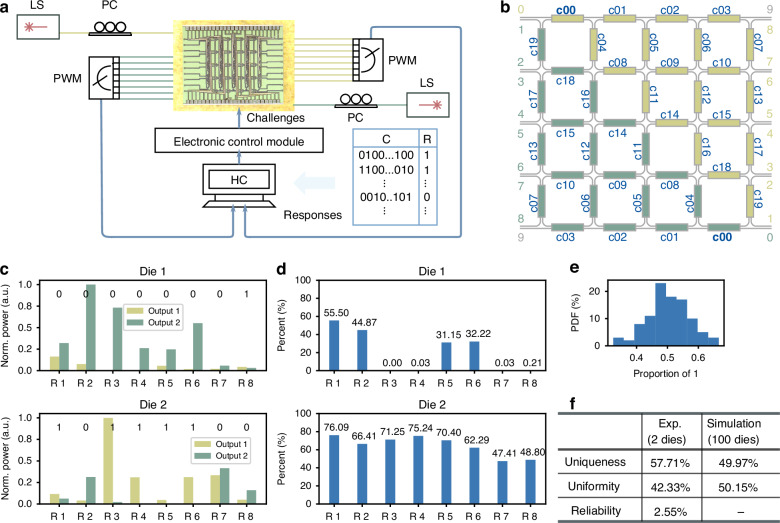
Experimental system and results for photonic PUFs (PPUF) implemented on LightIN. **a** Experiment system for PPUFs implemented on LightIN. **b** Programming topology of the rotational systematic PPUF. **c** Normalized output power and corresponding responses of two dies under the same challenge. **d** Proportion of “1” at different response bits of two dies under 128 challenges. **e** Probabilistic distribution of the proportion of “1” in responses of simulated PPUFs. **f** Summary of the uniqueness, uniformity, and reliability of the PPUF. LS laser source, PC polarization controller, PWM power meter, HC host computer

Inspired by the electronic arbiter PUFs, we establish an experimental setup as shown in Fig. 5a and propose a novel rotational-symmetric photonic PUF structure design as in Fig. 5b, where two theoretically equal-power lights are injected into the diagonal-position input port 0 (dark green) and 0 (light green). Challenges $C$ are represented by the programming voltages, where ‘1’ corresponds to the high-level voltage and ‘0’ to the low-level voltage, theoretically associated with the cross and bar states, respectively. Voltages are the same applied to the MZIs at equal logical positions with the same indices but different colors, as shown in Fig. 5b. Theoretically, since the structure is rotationally symmetric, the light powers from the corresponding output ports are equal. However, the output powers will deviate due to the process variations, the precision of the programming voltages, and environmental noise. Furthermore, the recirculating mesh enhances the uncertainty of the PPUFs. We define the response ${r}_{i}=1$ if the output port ${o}_{i,1}\ge {o}_{i,2}$, otherwise ${r}_{i}=$0, where $i$ is the $i$-th bit in the response $R$.

The performance of a PUF is typically evaluated using three key metrics: uniqueness (${UQ}$), uniformity (${UF}$), and reliability (${RL}$)^51^. The uniqueness is evaluated by the inter-die Hamming distance (HD), i.e.,

2 $${UQ}=\frac{2}{N(N-1)}\mathop{\sum }\limits_{i=1}^{N-1}\mathop{\sum }\limits_{j=i+1}^{N}{HD}({R}_{i},{R}_{j})/\left|R\right|$$

where ${N}$ is the number of chip dies, ${R}_{i}$ and ${R}_{j}$ are responses to the die ${i}$ and $j$, and ${|R|}$ is the response bit length. It represents the difference in response when the same challenges are applied to two PUF chips. The optimal theoretical ${UQ}$ value is 50%. Due to the chip number limits, we only tested two dies with 128 challenges by experiments. The inter-die Hamming difference between the two dies is 57.71%. Furthermore, we simulate 100 dies, in which the initial length differences between two arms in every MZI follow a Gaussian distribution ${\mathscr{N}}({\rm{\mu }}=-0.08{{\rm{e}}}^{-6},{\rm{\sigma }}=0.11{{\rm{e}}}^{-6})$, whose parameters are from the characterization results of prototype processing systems (detailed in the supplementary information note 8). Figure 5c presents the outputs and corresponding responses of two dies under the same challenge. The inter-die Hamming difference between 100 simulated dies is 49.97%, demonstrating the PUF’s uniqueness.

The uniformity is assessed by the proportion of “0” and “1” of the responses for one die with

3 $${UF}=\frac{1}{M}\mathop{\sum }\limits_{i=1}^{M}{R}_{i}/\left|R\right|$$

where $M$ is the number of different challenges applied and ${R}_{i}$ is the corresponding responses to the challenge ${C}_{i}$. Higher uniformity indicates better randomness in challenge-response pairs, which is essential for applications such as key generation and authentication. According to the definition, ${UF}$'s optimal value is 50%. Figure 5d shows the “1” proportions in different response bits of 128 challenges. The experimental results show that the average proportions of “1” for the two chips is 42.62%. In addition, the average uniformity for 100 simulated dies is 50.15% with their probabilistic distribution of the proportion of “1” as in Fig. 5e, demonstrating promising randomness.

Reliability is the reproduction of the same response from the same challenge under multiple measurements, evaluated by the intra-die Hamming distance with

4 $${RL}=\frac{1}{N}\mathop{\sum }\limits_{i=1}^{N}\mathop{\sum }\limits_{j=1}^{M}{HD}({R}_{i},{R}_{i,j})/\left|R\right|$$

where $N$ is the number of challenge-response pairs, ${M}$ is the measurement times, and ${R}_{i}$ and ${R}_{i,j}$ are responses to the challenge ${C}_{i}$ under the reference and the $j$-th measurement. We have tested the PUF for 10 times with 128 challenges. The intra-die HD of their responses is 2.55%, which is close to 0%. It should be noted that the intra-die HD between the nominal and other temperature conditions increases to over 10%. While this temperature sensitivity impacts the PUF reliability, it extends its functions to the PUF sensor and random number generation^50^. The experimental and simulation results (summarized in Fig. 5f) demonstrate it is a promising structure for photonic PUF design, addressing the encryption requirements of AI clusters in the optical domain.

## Discussion

While the LightIN has achieved diverse functions, we still find the current limitations and propose potential improvements for future work.

### Component-level optimization

The current single-phase-shifter MZI suffers from arm imbalance, unitary operation expression limits, and high-power consumption with configuration voltage up to $2{{\rm{V}}}_{{\rm{\pi }}}$ (${{\rm{V}}}_{{\rm{\pi }}}$ is the voltage to realize ${\rm{\pi }}$ phase shift). To address these issues, three additional phase shifters—one on the parallel arm and two at the input ports–can be added to the MZI, enabling precise phase control between two arms within the ${{\rm{V}}}_{{\rm{\pi }}}$ range and allowing for arbitrary unitary transformations^46^. Incorporating etch trenches can reduce heat dispersion, reducing ${{\rm{V}}}_{{\rm{\pi }}}$ and improving energy efficiency. Moreover, the MZI electrode can be optimized by closing it to the phase shifter waveguide, optimizing its geometric shape (e.g., enhancing the width and reducing the length), or utilizing alternative materials for a lower resistance value. It helps reduce ${{\rm{V}}}_{{\rm{\pi }}}$ for higher energy efficiency and enables shortening the phase shifter waveguide to reduce the MZI dimension. Asymmetrical directional couplers (ADC) will replace the current directional couplers in MZIs to achieve large bandwidths and low insertion loss. Additionally, edge couplers will replace the current grating couplers to support high-speed modulation signals in optical computing and switching applications.

As for insertion loss, reducing waveguide propagation loss is a critical pathway for minimizing insertion loss. This can be achieved through advanced fabrication techniques such as thermal oxidation^52^ and chemical polishing^53^ to minimize sidewall roughness, which directly reduces scattering losses. Additionally, employing wider waveguides in the design phase can further mitigate propagation losses by enhancing mode confinement and reducing edge scattering^54,55^.

### Circuit-level enhancement

While the waveguide lengths among the MZIs in the square mesh are designed to be equal, the waveguide lengths between the input/output gratings and the MZIs are not uniform and cannot be calibrated due to the lack of phase shifters. This inconsistency causes temporal misalignment among parallel input signals, particularly affecting computing precision and speed in optical computing applications^56^. Future designs will incorporate equal waveguide lengths and additional phase shifters for precise path control.

Regarding the scalability, utilizing the optimized MZIs with smaller dimensions and lower loss, we expect to realize large-scale integrated P-FPGAs for more diverse functions and higher performance (e.g., larger computation throughput, which is proportional to the P-FPGA size N^2^). Based on the optimized devices from a mature Process Design Kit (PDK), implementing a 32 × 32 P-FPGA is feasible. A 64 × 64 P-FPGA would be achievable once the accumulated propagation losses are further reduced, e.g., those from the ADCs. The comprehensive analysis for the P-FPGA size and its performance is presented in the supplementary information note 9.

Besides, in the current LightIN, although we have reduced the number of electronic pads by integrating a single-arm heater on the MZI and sharing the ground pad for multiple heaters, the photonic chip still exhibits a footprint of 3.8 × 3 mm². This large footprint limits its scalability in space-constrained scenarios. To address this, future iterations will adopt the 2.5D hybrid advanced packaging approach with flip-chip bonding to connect the electronic control circuit with the on-chip photonic components, potentially reducing the footprint by over 55%^57^. In other words, in a fixed footprint, the optimized MZIs and flip-chip bonding scheme can realize a larger-scale integration P-FPGA.

To further enhance system scalability with limited-size chips, the mesh structure can be modified by disconnecting the horizontal MZIs at the first and last rows (instead of connecting them with straight waveguides as in the demonstrated design) and adding input/output ports similar to the vertical MZIs at the first and last column. This modification supports multiple chips (or cores) with such a mesh structure to be directly connected and expanded into a large mesh through fiber or the lens coupling package. (A diagram of the structure modification and expansion is presented in the supplementary information note 10). This expansion requires limited updates to the TCA framework, as the fundamental architecture remains unchanged, with only the size increasing. This multi-core scalability offers a promising pathway to constructing large-scale photonic systems by seamlessly integrating photonic chiplets, enabling significantly enhanced processing capabilities and system flexibility.

### System integration and automation

In the current LightIN, the electronic control module is a multi-port voltage source with the host computer to run the TCA framework and store data and instructions, which makes the LightIN bulky and cumbersome. Integrating the TCA framework and voltage control into a single System-on-chip (SoC)-enabled FPGA, along with photonic chip packaging, will enable a more compact and efficient system^58^.

In terms of automation, challenges also exist. At the hardware level, the recirculating square mesh design inherently contains hardware redundancy to support multiple applications within a single chip. For instance, in the wavelength locking scenario discussed in the previous section, using only one differentiator in the 4 × 4 square mesh leaves many components inactive, appearing to be resource inefficient. However, this redundancy can be advantageous as these inactive components can be programmed or reconfigured for other functions. As demonstrated in the optical I/O applications, when multiple MRMs require wavelength locking, four differentiators can be efficiently programmed in the P-FPGAs (see supplementary information note 11). Moreover, when defects occur in either photonic circuits or electronic control circuits, the redundant available MZI resources and input/output ports can maintain the functionality by establishing alternative paths, which improves the chip’s fault tolerance and robustness^59^. At the software level, as the square mesh scales up, the predetermined method of topologies for different functions might be less effective in the TCA compilation phase. Therefore, our next-stage work will focus on developing an intelligent compilation flow for automatic resource allocation and fault tolerance.

### Future vision: photo-electronic AI cluster

The current AI clusters primarily rely on electronic chips such as GPUs and CPUs with optical modules for data transmission. While P-FPGAs offer advantages in speed and energy efficiency, their integration faces challenges due to the overhead from large amounts of optoelectronic conversions. To address this limitation, we envision hybrid photo-electronic AI clusters where PICs extend beyond data transmission while minimizing optoelectronic conversions^60^. For example, when a large amount of data is computed in the photonic chips of a distributed system, it can be transmitted and processed by the optical switching without optical-electronic-optical (OEO) conversions, since all data are in the optical domain. Furthermore, when the optical storage and controlling challenges are addressed, all-optical processing systems will be available. The photo-electronic AI clusters are continuously evolving.

However, before that, we need to develop more advanced PICs. Although recent research shows promising ASPICs capable of processing 128-to-256 parallel signals with over 10,000 photonic components^57,58^ and optical modules for 136-channel switching^61^, a considerable gap exists between current ASPIC hardware capabilities and the requirements for implementing evolving AI models. Before ASPIC design and fabrication, P-FPGAs can be configured for rapid application verification^23,30^. These P-FPGAs, interconnected through efficient data interfaces, have the potential to create an optimized system for high-speed and energy-efficient AI computing.

## Materials and methods

### Design, fabrication, and packaging of the silicon-integrated P-FPGAs

The photonic chip occupies a footprint of 3.8 × 3 mm^2^. It mainly consists of the grating couplers and the MZIs. Twenty grating couplers are equally distributed at the two opposite edges, spaced at 222.22 μm. The 40 MZIs connect as a square mesh with 450 nm-width silicon waveguides. Each MZI features a directional coupler with a length of 11.5 $\upmu$m and a gap of 200 nm, and has an arm length of 208 μm. A 100 μm-length heater is employed as the phase shifter, controlled by a pair of electronic pads. The heaters in one column share one ground pad. Therefore, the silicon photonic chip contains 49 pads arranged along two opposite edges, with 24 and 25 spaced at 152 μm and 145 μm intervals, respectively. The MZIs are arranged in a square mesh topology, with each side of the square unit measuring 500 μm. This configuration ensures that the distance between adjacent heaters exceeds 280 μm, which is sufficient to minimize thermal crosstalk between phase shifters. The chip is fabricated on a silicon-on-insulator (SOI) wafer consisting of a 2.2 μm-thick oxide (SiO_2_) cladding layer, a 220 nm-thick silicon (Si) layer, and a 2 μm-thick buried thermal oxide (SiO_2_) layer. Light couples through two fiber arrays to the grating couplers, while the electronic pads are connected to a PCB with wire bonding.

### Prototype system construction

In this section, we present the experimental setups and equipment used for implementing the aforementioned functions.

#### Computing acceleration in AI clusters

We establish two experimental systems to verify the computing acceleration functions implemented in the LightIN. The first system is a low-speed experiment system to validate the unitary and non-unitary matrix expressions of the chip. The second system is a high-speed experiment system to demonstrate the computing performance of the LightIN. In the low-speed experiment, a multi-channel laser source (SOUTHERN PHOTONICS, TLS150-20) provides 8 independent lights for the bi-directional unitary matrix-vector multiplications (4 for each direction) and 6 for the non-unitary ones. The light wavelengths are set to 1560 nm, and their powers are adjusted to 16 mW or 0 mW for the matrix expression test and other corresponding values to encode the Iris data for the NN test. The output optical signals are detected by a multi-channel power meter (KEYSIGHT, N7745A). In the high-speed experiment, the carriers are modulated via the intensity modulators (NOEIC, MZ1550-LN-40, bandwidth of over 28 GHz) by the AWG (KEYSIGHT, M8194A) with a sampling rate of 120 GSa/s and vertical resolution of 8 bits, and the multiplied signals are detected by a PD array (NOEIC, DR4-RX, bandwidth of over 30 GHz) and sampled by the oscilloscope (KEYSIGHT, UXR0594A) with a sampling rate of 256 GSa/s and vertical resolution of 10 bits.

#### Signal processing for optical I/O

To verify the LightIN functions in signal processing to realize wavelength-locking for MRMs, an experimental system, as shown in Fig. 3a, is established. A single-wavelength laser (SOUTHERN PHOTONICS, TLS150-20) is set to 1555 nm and 16 mW as the carrier. The light is injected into a self-developed MRM^62^ and modulated by the RF signal generated from an AWG (KEYSIGHT, M8194A). The modulated signal is then amplified by an EDFA (Amonics, AEDFA-CL-20-R-FC) and split into two paths, one for the eye diagram (KEYSIGHT, DCA-M N1092A) to demonstrate the signal quality and the other directed to the LightIN-based control loop for optimum heater voltage searching. The optical signal output from the LightIN is detected by the power meter (KEYSIGHT, N7745A). EDFAs, compensating for the coupling loss, can be removed when the photonic components for the automatic locking are integrated with MRMs in one chip.

#### Optical switching in AI clusters

To demonstrate the LightIN performance as an optical switcher, we use a broadband light source (Realphoton, ASE-B-F-CL-50-S-FA) and an optical spectrometer (YOKOGAWA, AQ6370D) to observe the transmission spectrums between any two input and output ports.

#### Secure encryption for AI clusters

In this prototype system, two independent single-wavelength lasers (SOUTHERN PHOTONICS, TLS150-20) are set to 1560 nm. The two laser powers are 12.5 dBm and carefully calibrated by the polarization controllers (PC) between the laser source and the P-FPGA to maintain the injection power equality according to the PPUF design principle. The host computer generates the challenge bits, stimulates the PPUF via the multi-channel voltage source (NOEIC, MCVS-128C), and reads and compares the PPUF output powers through the multi-channel power meter (KEYSIGHT, N7745A) to obtain the responses.

## Supplementary information

Supplementary Information

## Data Availability

The data that support the findings of this study are available from the corresponding author on reasonable request.

## References

[1] Chang, Y. P. et al. A survey on evaluation of large language models. *ACM Trans. Intell. Syst. Technol.* **15**, 39 (2024).

[2] Moor, M. et al. Foundation models for generalist medical artificial intelligence. *Nature* **616**, 259–265 (2023). 37045921 10.1038/s41586-023-05881-4

[3] Zhang, K. P. et al. AI-TP: Attention-based interaction-aware trajectory prediction for autonomous driving. *IEEE Trans. Intell. Veh.* **8**, 73–83 (2022).

[4] Zhang, Z. et al. Structured light meets machine intelligence. *eLight* **5**, 26 (2025).

[5] Wu, N. et al. Intelligent nanophotonics: When machine learning sheds light. *eLight* **5**, 5 (2025).

[6] Pan, S. et al. Facebook’s tectonic filesystem: Efficiency from exascale. *Proceedings of the 19th USENIX Conference on File and Storage Technologies*. Santa Clara, CA, USA: USENIX, 2021, 217-231.

[7] Zhao, M. et al. Understanding data storage and ingestion for large-scale deep recommendation model training: industrial product. *Proceedings of the 49th Annual International Symposium on Computer Architecture*. New York, NY, USA: ACM, 2022, 1042–1057.

[8] Yang, Y., Liu, K., Gao, Y., Wang, C. & Cao, L. Advancements and challenges in inverse lithography technology: A review of artificial intelligence-based approaches. *Light Sci. Appl.* **14**, 250 (2025). 10.1038/s41377-025-01923-w PMC12287475 40701983

[9] Morik, K. & Marwedel, P. Machine learning under resource constraints-fundamentals. (Berlin: Walter de Gruyter GmbH & Co KG, 2022).

[10] Gholami, A. et al. Ai and memory wall. *IEEE Micro* **44**, 33–39 (2024).

[11] Zhao, C. G. et al. Insights into DeepSeek-V3: scaling challenges and reflections on hardware for AI architectures. *Proceedings of the 52nd Annual International Symposium on Computer Architecture*. Tokyo, Japan: ACM, 2025, 1731-1745.

[12] Jacobs, R. et al. A practical guide to machine learning interatomic potentials–status and future. *Curr. Opin. Solid State Mater. Sci.* **35**, 101214 (2025).

[13] Mehonic, A. & Kenyon, A. J. Brain-inspired computing needs a master plan. *Nature* **604**, 255–260 (2022). 35418630 10.1038/s41586-021-04362-w

[14] Filipovich, M. J. et al. Silicon photonic architecture for training deep neural networks with direct feedback alignment. *Optica* **9**, 1323–1332 (2022).

[15] Liu, H. et al. A 4×112 Gb/s PAM-4 silicon-photonic transmitter and receiver chipsets for linear-drive co-packaged optics. *IEEE J. Solid-State Circuits* **59**, 3263–3276 (2024).

[16] Shi, Y. C. et al. Silicon photonics for high-capacity data communications. *Photonics Res.* **10**, A106–A134 (2022).

[17] Ruan, Z. S. et al. Flexible orbital angular momentum mode switching in multimode fibre using an optical neural network chip[J]. *Light Adv. Manuf.* **5**, 23 (2024).

[18] Seok, T. J. et al. Wafer-scale silicon photonic switches beyond die size limit. *Optica* **6**, 490–494 (2019).

[19] Huang, C. R. et al. A silicon photonic–electronic neural network for fibre nonlinearity compensation. *Nat. Electron.* **4**, 837–844 (2021).

[20] Shastri, B. J. et al. Photonics for artificial intelligence and neuromorphic computing. *Nat. Photonics* **15**, 102–114 (2021).

[21] Ashtiani, F., Geers, A. J. & Aflatouni, F. An on-chip photonic deep neural network for image classification. *Nature* **606**, 501–506 (2022). 35650432 10.1038/s41586-022-04714-0

[22] Chen, Z. & Segev, M. Highlighting photonics: Looking into the next decade. *eLight* **1**, 2 (2021).

[23] Shekhar, S. et al. Roadmapping the next generation of silicon photonics. *Nat. Commun.* **15**, 751 (2024). 38272873 10.1038/s41467-024-44750-0 PMC10811194

[24] Wade, M. et al. Driving compute scale-out performance with optical I/O chiplets in advanced system-in-package platforms. *2023 IEEE Hot Chips 35 Symposium (HCS)*. Palo Alto, CA, USA: IEEE, 2023, 1.

[25] Yu, X. et al. Parallel optical computing capable of 100-wavelength multiplexing. *eLight* **5**, 10 (2025).

[26] Zhu, S. & Zhu, N. H. Nonlinear optoelectronic engine drives monolithic integrated photonic computing. *Light Sci. Appl.* **14**, 302 (2025). 10.1038/s41377-025-01970-3 PMC12411629 40908283

[27] Oguz, I. et al. Resource-efficient photonic networks for next-generation AI computing. *Light Sci. Appl.* **14**, 34 (2025). 10.1038/s41377-024-01717-6 PMC11698740 39753539

[28] Xu, Z. H. et al. Large-scale photonic chiplet Taichi empowers 160-TOPS/W artificial general intelligence. *Science* **384**, 202–209 (2024). 38603505 10.1126/science.adl1203

[29] Bandyopadhyay, S. et al. Single-chip photonic deep neural network with forward-only training. *Nat. Photonics* **18**, 1335–1343 (2024).

[30] Pérez, D. et al. Multipurpose silicon photonics signal processor core. *Nat. Commun.* **8**, 636 (2017). 28935924 10.1038/s41467-017-00714-1 PMC5608755

[31] Bogaerts, W. et al. Programmable photonic circuits. *Nature* **586**, 207–216 (2020). 33028997 10.1038/s41586-020-2764-0

[32] Zhang, Q., Zhang, Y. & Czarske, J. FPGA-accelerated mode decomposition for multimode fiber-based communication. *Light Adv. Manuf.* **6**, 272–283 (2025).

[33] Reck, M. et al. Experimental realization of any discrete unitary operator. *Phys. Rev. Lett.* **73**, 58–61 (1994). 10056719 10.1103/PhysRevLett.73.58

[34] Clements, W. R. et al. Optimal design for universal multiport interferometers. *Optica* **3**, 1460–1465 (2016).

[35] Perez, D. et al. Silicon photonics rectangular universal interferometer. *Laser Photonics Rev.* **11**, 1700219 (2017).

[36] Wang, J. W. et al. Integrated photonic quantum technologies. *Nat. Photonics* **14**, 273–284 (2020).

[37] Shen, Y. C. et al. Deep learning with coherent nanophotonic circuits. *Nat. Photonics* **11**, 441–446 (2017).

[38] Annoni, A. et al. Unscrambling light—automatically undoing strong mixing between modes. *Light Sci. Appl.* **6**, e17110 (2017). 30167222 10.1038/lsa.2017.110 PMC6062024

[39] Choutagunta, K. et al. Adapting Mach–Zehnder mesh equalizers in direct-detection mode-division-multiplexed links. *J. Lightwave Technol.* **38**, 723–735 (2020).

[40] Zhuang, L. M. et al. Programmable photonic signal processor chip for radiofrequency applications. *Optica* **2**, 854–859 (2015).

[41] Pérez-López, D. et al. General-purpose programmable photonic processor for advanced radiofrequency applications. *Nat. Commun.* **15**, 1563 (2024). 38378716 10.1038/s41467-024-45888-7 PMC10879507

[42] Zhao, M. Y., Wu, B. & Dong, J. J. On-chip multifunctional self-configurable quadrilateral MZI network. *Opt. Mater. Expr.* **13**, 3138–3147 (2023).

[43] Zhang, H. et al. An optical neural chip for implementing complex-valued neural network. *Nat. Commun.* **12**, 457 (2021). 33469031 10.1038/s41467-020-20719-7 PMC7815828

[44] Pai, S. et al. Experimentally realized in situ backpropagation for deep learning in photonic neural networks. *Science* **380**, 398–404 (2023). 37104594 10.1126/science.ade8450

[45] Tischler, N., Rockstuhl, C. & Słowik, K. Quantum optical realization of arbitrary linear transformations allowing for loss and gain. *Phys. Rev. X* **8**, 021017 (2018).

[46] Hamerly, R. et al. Toward the information-theoretic limit of programmable photonics. *APL Photonics* **10**, 110803 (2025).

[47] Wade, M. et al. TeraPHY: a chiplet technology for low-power, high-bandwidth in-package optical I/O. *IEEE Micro* **40**, 63–71 (2020).

[48] Cheng, Q. X. et al. Silicon photonic switch topologies and routing strategies for disaggregated data centers. *IEEE J. Sel. Top. Quantum Electron.* **26**, 8302010 (2020).

[49] Wang, R. P. et al. Recent advances of volatile memristors: devices, mechanisms, and applications. *Adv. Intell. Syst.* **2**, 2000055 (2020).

[50] Gao, Y. S., Al-Sarawi, S. F. & Abbott, D. Physical unclonable functions. *Nat. Electron.* **3**, 81–91 (2020).

[51] Athanas, P., Pnevmatikatos, D. & Sklavos, N. Embedded systems design with FPGAs. (New York: Springer, 2013).

[52] Lee, K. K. et al. Fabrication of ultralow-loss Si/SiO2 waveguides by roughness reduction. *Opt. Lett.* **26**, 1888–1890 (2001). 18059727 10.1364/ol.26.001888

[53] Shi, X. D. et al. Wet-oxidation-assisted chemical mechanical polishing and high-temperature thermal annealing for low-loss 4H-SiC integrated photonic devices. *Materials* **16**, 2324 (2023). 36984202 10.3390/ma16062324 PMC10058445

[54] Xie, Y. W. et al. Complex-valued matrix-vector multiplication using a scalable coherent photonic processor. *Sci. Adv.* **11**, eads7475 (2025). 40184444 10.1126/sciadv.ads7475 PMC11970466

[55] Hong, S. H. et al. Versatile parallel signal processing with a scalable silicon photonic chip. *Nat. Commun.* **16**, 288 (2025). 39746962 10.1038/s41467-024-55162-5 PMC11695732

[56] Zhu, Y. et al. Silicon photonic neuromorphic accelerator using integrated coherent transmit-receive optical sub-assemblies. *Optica* **11**, 583–594 (2024).

[57] Hua, S. Y. et al. An integrated large-scale photonic accelerator with ultralow latency. *Nature* **640**, 361–367 (2025). 40205213 10.1038/s41586-025-08786-6 PMC11981923

[58] Ahmed, S. R. et al. Universal photonic artificial intelligence acceleration. *Nature* **640**, 368–374 (2025). 40205212 10.1038/s41586-025-08854-x

[59] Aghaee Rad, H. et al. Scaling and networking a modular photonic quantum computer. *Nature* **638**, 912–919 (2025). 39843755 10.1038/s41586-024-08406-9 PMC11864973

[60] Li, R. J. et al. Photonics for neuromorphic computing: Fundamentals, devices, and opportunities. *Adv. Mater.* **37**, 2312825 (2025). 10.1002/adma.202312825 39011981

[61] Jouppi, N. et al. TPU v4: An optically reconfigurable supercomputer for machine learning with hardware support for embeddings. *Proceedings of the 50th Annual International Symposium on Computer Architecture*. Orlando, FL, USA: ACM, 2023, 82.

[62] Zhang, Y. G. et al. 240 Gb/s optical transmission based on an ultrafast silicon microring modulator. *Photonics Res.* **10**, 1127–1133 (2022).

