# Supplementary material for: LightIN: a versatile silicon-integrated photonic field programmable gate array with an intelligent configuration framework for next-generation AI clusters
Source: Light Sci Appl. 2026 Mar 11;15:165. doi: 10.1038/s41377-026-02209-5 (PMC12979840; doi:10.1038/s41377-026-02209-5)
Supplement: Supplementary file 1 — Supplementary Information [file 41377_2026_2209_MOESM1_ESM.docx]

Supplementary Information for

**LightIN: a versatile silicon-integrated photonic field programmable gate array with an intelligent configuration framework for next-generation AI clusters**

Ying Zhu^1,+^, Yifan Liu^1,+^, Xinyu Yang^1^, Kailai Liu^1,2^, Xin Hua^1^, Ming Luo^2^, Jia Liu^1^, Siyao Chang^1^, Jie Yan^1^, Shengxiang Zhang^1^, Miao Wu^1^, Zhicheng Wang^1^, Hongguang Zhang^1^, Dong Wang^1^, Daigao Chen^1^, Xi Xiao^1,3,*^, Shaohua Yu^3^

^1^ National Information Optoelectronics Innovation Center, China Information and Communication Technologies Group Corporation, Youkeyuan Road 88, Wuhan 430074, Hubei, China

^2^ State Key Laboratory of Optical Communication Technologies and Networks, China Information and Communication Technologies Group Corporation, Gaoxinsi Road 6, Wuhan 430074, Hubei, China

^3^ Peng Cheng Laboratory, Shahexi Road 6001, Shenzhen 518108, Guangdong, China

^*^Corresponding author: Xi Xiao, [xiaoxi@noeic.com](mailto:xiaoxi@noeic.com)

^+^These authors contributed equally to this work.

[**Supplementary Information Note 1:** An example of the MZI characterization result. 3](#_Toc218773766)

[**Supplementary Information Note 2:** Comparison of footprint efficiency and insertion loss between square mesh and hexagon mesh structures. 4](#_Toc218773767)

[**Supplementary Information Note 3**: Energy efficiency analysis for LightIN. 6](#_Toc218773768)

[**Supplementary Information Note 4:** The mathematical derivation for realizing a non-unitary transformation matrix using the diamond mesh based on unitary MZI structures. 8](#_Toc218773769)

[**Supplementary Information Note 5:** Advantage analysis for the diamond structure compared to alternative architectures. 11](#_Toc218773770)

[**Supplementary Information Note 6:** Wavelength locking approaches: electronic vs. photonic solutions. 13](#_Toc218773771)

[**Supplementary Information Note 7:** Path-independent loss (PILOSS) non-blocking switch in the MZI-based recirculating square mesh. 16](#_Toc218773772)

[**Supplementary Information Note 8:** Measurement and analysis for the initial phase shift distribution in the MZI-based square meshes. 17](#_Toc218773773)

[**Supplementary Information Note 9:** A quantitative analysis for the potential scale of the MZI-based square mesh. 18](#_Toc218773774)

[**Supplementary Information Note 10:** The modification and expansion of the square mesh core to realize multi-core large-scale integration. 21](#_Toc218773775)

[**Supplementary Information Note 11:** A possible topology for LightIN to simultaneously lock wavelengths of four micro-ring modulators. 22](#_Toc218773776)

[Reference 23](#_Toc218773777)

**Supplementary Information Note 1:** An example of the MZI characterization result.

Following the method proposed in **Testing: MZI characterization**, we obtain the output intensity varying with the applied power (the applied voltage × the measured current) of an MZI on the photonic chip, as in Figure S1(a), and further derive its phase shifts varying with the applied power, as in Figure S1(b). It should be noted that when measuring the Bar output port (where the output port corresponds to the input port position), if the intensity initially increases with the applied power, the initial phase shift is in the range of $\left[ 0 \right.,\left. \pi\right]$, otherwise $\left[ -\pi\right.,\left. 0 \right]$. When measuring the Cross output port, the situation is opposite according to equation (1) in the manuscript. The presented results confirm that the tuning range of the phase shifter on the MZI can achieve a complete 2$\pi$ range. Within this range, the phase shift increases linearly with the applied power. Accordingly, we can establish the table of the phase varying with applied voltage, ensuring the realization of reliable MZI configurations for various applications.


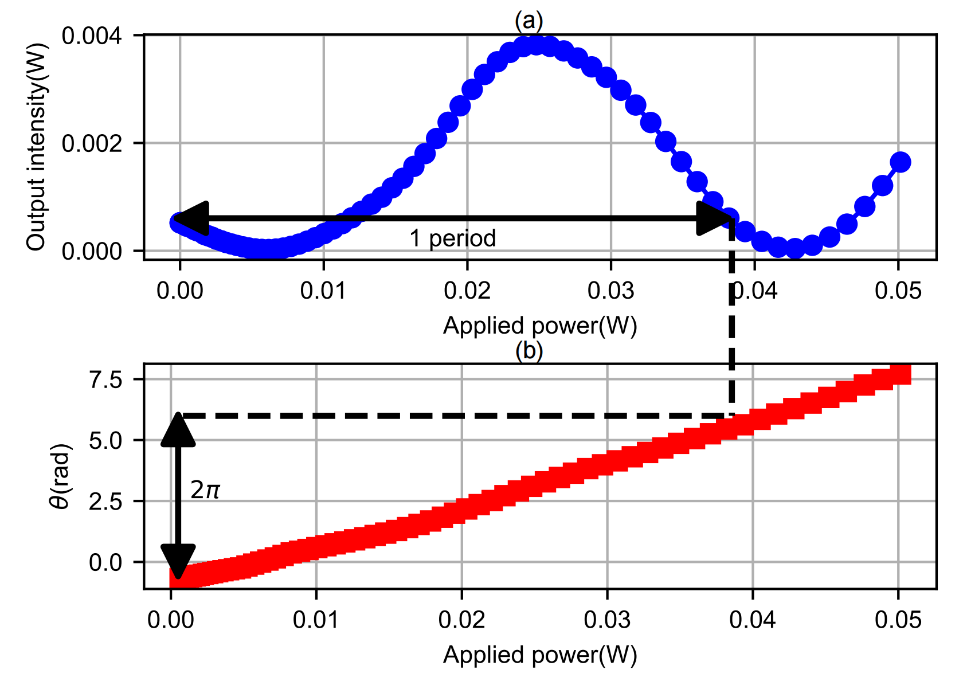


Figure S1 The output intensity and phase shift vary with the applied power.

**Supplementary Information Note 2:** Comparison of footprint efficiency and insertion loss between square mesh and hexagon mesh structures.

The square and hexagon recirculating meshes can provide loop paths through programming, enhancing the application range of photonic programmable chips. Compared to the widely studied hexagon form, the square form hasn’t been fully explored. Here, we analyze these two structures based on identical programmable unit cell MZIs and demonstrate the advantages of square mesh in terms of footprint efficiency and insertion loss.

Figure S2 illustrates the implementations of square and hexagon meshes for 4×4 unitary matrix computation. The side lengths of the minimal square and hexagon in both meshes are defined as $L$, thus the radii of bending waveguides in the hexagon and square meshes satisfy the relationship $R_{h}=\sqrt{3}R_{s}$. For a 4×4 unitary matrix operation, the hexagon mesh requires six hexagons with a total footprint of $9\sqrt{3}L^{2}$. The square mesh utilizes sixteen squares to complete two 4×4 unitary matrices with an average footprint ${8L}^{2}$ for each. Thus, the operations per footprint unit are $1/{9\sqrt{3}L^{2}}$ and $1/{8L^{2}}$ for hexagon and square meshes, respectively. Therefore, the footprint efficiency of the square mesh is improved by 1.95× compared to hexagon mesh.


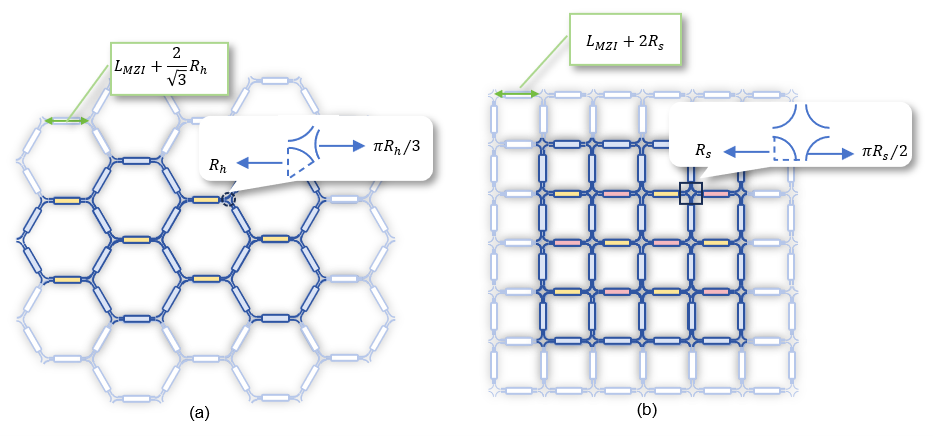


Figure S2 The implementation of 4×4 unitary matrices in the hexagon and square meshes.

For the insertion loss, to complete the 4×4 unitary operations, the signal paths in both forms include nine MZIs and ten bending waveguides. When the same programmable unit cells (i.e., MZIs in our demonstration) are applied to both forms, the insertion loss differences mainly come from bending waveguides to connect MZIs. With our implemented bending radius ($\geq80 \mu m$, above the 20 $\mu m$ threshold), diffraction losses are negligible. The insertion loss is dominated by the waveguide length. The length of the ${90}^{^{\circ}}$ bending waveguide in the square mesh is ${\pi R_{s}}/2$, which is slightly shorter than the ${60}^{^{\circ}}$ waveguide with a length of ${\pi R_{h}}/3={\sqrt{3}\pi R_{s}}/3$ in the hexagon mesh. Therefore, the insertion loss of the square mesh is lower than that of the hexagon mesh.

**Supplementary Information Note 3**: Energy efficiency analysis for LightIN.

The total energy consumption for the P-FPGA system consists of two main parts: the photonic integrated circuits (PICs) and the electronic integrated circuits (EICs).

For the PICs (i.e., the P-FPGA core), the energy consumption mainly comes from the 40 MZIs. To achieve a $\pi$-shift on a phase shifter, an averaged 3 *V* voltage is required. The resistor for a phase shifter is 100 $\Omega$. According to mathematical analysis in ^1^, the expectation of phase shifts $\theta$ is $\pi$ (Note: While the phase shifts $\theta$ under the belongs to $\left[ -\pi,\pi\right]$ in mathematical derivations with an expectation of 0 as in the original reference, in our scenario $\theta$ belongs to $[0,2\pi]$ due to the MZI with only one-arm phase shift.)

Therefore, the average energy consumption for each MZI is ${3\times3}/{100}=0.09 J s^{-1}$. With a computing speed of 1.92 *TOPS* ((ComplexMatrix_4×4_×RealValuedVector_4×1_ in P-FPGA + Squared Addition in photodetectors (PDs)) × 2 directions × 10 *GBaud* = 1.92 *TOPS*), the energy efficiency of P-FPGA is calculated as $40*{0.09}/{1.92}=1.875 pJ {MAC}^{-1}$.

The EICs in the P-FPGA system are responsible for multiple functions, including data storage, transmission, instruction handling, and signal conditioning. In our current experimental setup, these functions are implemented through a combination of a host computer, an electronic control module, and some testing devices. However, this setup has not been specifically optimized for integration and application, making its energy efficiency assessment less meaningful for the actual P-FPGA system.

A highly integrated system that combines these function modules with the PIC on a single board shows promise for achieving $1\sim10 pJ {MAC}^{-1}$. For reference, the Photonic Arithmetic Computing Engine (PACE) ^2^ successfully demonstrates not only a system-in-package integration of both PIC and EIC but also a system-on-board integration of the P/EIC and storage and transmission modules. PACE can control more than 4000 programmable photonic MZIs in the PICs and support 64-channel data loading and retrieval, which satisfy our P-FPGA’s requirements. It has achieved a computing speed of 8 *TOPS* and an energy efficiency of around $0.42 pJ {MAC}^{-1}$. To run the TCA framework on board, we require a processor core, such as utilizing the one in the AMD UltraScale+ FPGA, which will increase the system’s typical energy consumption by below 10 W (depending on algorithm complexity). While the board integration design would lead to reduced data modulation speed, affecting the computing speed for the 4×4 P-FPGA, we can achieve a higher overall computing speed of over 3.2 *TOPS* (2×40×40×1 *G*) by scaling the P-FPGA to 40×40 (the total MZI number is 3280, below 4000). The potential energy efficiency can achieve $3 pJ {MAC}^{-1}$. Additionally, the TCA framework overhead is part of the EIC energy consumption. Its impact on overall system efficiency largely depends on the duration of the implemented function. For example, in scenarios where the P-FPGA serves as an optical switch in the AI cluster with prolonged switching operations, the TCA framework overhead becomes negligible. This suggests that a similar integration design approach could be adopted for our P-FPGA system to achieve board-level integration with comparable energy efficiency.

**Supplementary Information Note 4:** The mathematical derivation for realizing a non-unitary transformation matrix using the diamond mesh based on unitary MZI structures.

We take an example of a 3x3 non-unitary matrix realized by a 6-input-6-output diamond structure.


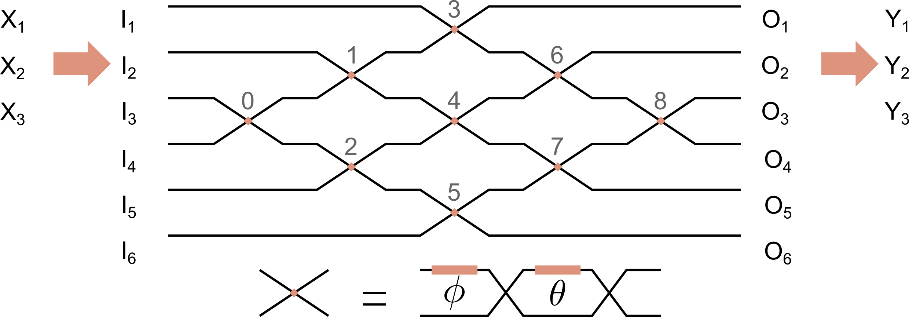


Figure S3 The 6-input-6-output diamond structure based on MZIs.

Notably, the diamond structure is a passive mesh. Therefore, the 3x3 non-unitary transformation matrix from its 3 inputs to 3 outputs and the 6x6 unitary transformation matrix from its complete 6 inputs to 6 outputs are contracting or preserving matrices, meaning their eigenvalues’ modules are no greater than 1. While it is a limit of the diamond structure, we can realize an expanding matrix based on the diamond structure by adding parallel amplifiers before or after the structure, or by having the optical intensities that represent the multiplication results numerically correspond to values that expand the matrix.

First, for an MZI at the position of *i/i+1* row in the mesh, its transformation matrix is

$T_{MZI(i,i+1)} = \left( \begin{matrix} \begin{matrix} 1 & 0 & \cdots\\ 0 & \ddots& \cdots\\ \vdots& \cdots& {ie}^{i\theta/2}e^{i\varphi} \sin\frac{\theta}{2} \end{matrix} & \begin{matrix} \cdots& \cdots& 0 \\ \cdots& \cdots& \vdots\\ {ie}^{i\theta/2}\cos\frac{\theta}{2} & \cdots& \vdots\end{matrix} \\ \begin{matrix} \vdots& \cdots& {ie}^{i\theta/2}e^{i\varphi} \cos\frac{\theta}{2} \\ \vdots& \cdots& \cdots\\ 0 & \cdots& \cdots\end{matrix} & \begin{matrix} {-ie}^{i\theta/2}\sin\frac{\theta}{2} & \cdots& \vdots\\ \cdots& \ddots& \vdots\\ \cdots& \cdots& 1 \end{matrix} \end{matrix} \right)$, (1)

where the non-zero and non-one values are at the (row, column)=(*i, i*),(*i, i+1*),(*i+1,i*), and (*i+1, i+1*).

For the diamond structure, we can consider that the transformation matrix from the inputs to the positions before the MZI 0 is an identity matrix as

$T_{0}=\left( \begin{matrix} \begin{matrix} 1 & 0 & 0 \\ 0 & 1 & 0 \\ 0 & 0 & 1 \end{matrix} & \begin{matrix} 0 & 0 & 0 \\ 0 & 0 & 0 \\ 0 & 0 & 0 \end{matrix} \\ \begin{matrix} 0 & 0 & 0 \\ 0 & 0 & 0 \\ 0 & 0 & 0 \end{matrix} & \begin{matrix} 1 & 0 & 0 \\ 0 & 1 & 0 \\ 0 & 0 & 1 \end{matrix} \end{matrix} \right)$. (2)

According to the MZI transformation matrix in (1), MZI 0 introduces a rotation to the elements of the 3rd and 4th rows, resulting in the transformation matrix shown below:

$T_{1,2}=\left( \begin{matrix} \begin{matrix} 1 & 0 & 0 \\ 0 & 1 & 0 \\ 0 & 0 & * \end{matrix} & \begin{matrix} 0 & 0 & 0 \\ 0 & 0 & 0 \\ * & 0 & 0 \end{matrix} \\ \begin{matrix} 0 & 0 & * \\ 0 & 0 & 0 \\ 0 & 0 & 0 \end{matrix} & \begin{matrix} * & 0 & 0 \\ 0 & 1 & 0 \\ 0 & 0 & 1 \end{matrix} \end{matrix} \right)$, (3)

where * represents the non-zero and non-one values, simplifying the expression.

MZI 1 and MZI 2 introduce rotations to the elements of rows 2, 3, and 4. After these operations, the transformation matrix becomes

$T_{3,4,5}=\left( \begin{matrix} \begin{matrix} 1 & 0 & 0 \\ 0 & * & * \\ 0 & * & * \end{matrix} & \begin{matrix} 0 & 0 & 0 \\ * & 0 & 0 \\ * & 0 & 0 \end{matrix} \\ \begin{matrix} 0 & 0 & * \\ 0 & 0 & * \\ 0 & 0 & 0 \end{matrix} & \begin{matrix} * & * & 0 \\ * & * & 0 \\ 0 & 0 & 1 \end{matrix} \end{matrix} \right)$. (4)

MZI 3, 4, and 5 further rotate the elements in all rows, updating the transformation matrix to

$T_{6,7}=\left( \begin{matrix} \begin{matrix} * & * & * \\ * & * & * \\ 0 & * & * \end{matrix} & \begin{matrix} * & 0 & 0 \\ * & 0 & 0 \\ * & * & 0 \end{matrix} \\ \begin{matrix} 0 & * & * \\ 0 & 0 & * \\ 0 & 0 & * \end{matrix} & \begin{matrix} * & * & 0 \\ * & * & * \\ * & * & * \end{matrix} \end{matrix} \right)$. (5)

After the MZI 6 and 7, the transformation matrix is obtained as follows:

$T_{8}=\left( \begin{matrix} \begin{matrix} * & * & * \\ * & * & * \\ * & * & * \end{matrix} & \begin{matrix} * & 0 & 0 \\ * & * & 0 \\ * & * & 0 \end{matrix} \\ \begin{matrix} 0 & * & * \\ 0 & * & * \\ 0 & 0 & * \end{matrix} & \begin{matrix} * & * & * \\ * & * & * \\ * & * & * \end{matrix} \end{matrix} \right)$. (6)

Eventually, after the MZI 8, the complete unitary transformation matrix of the diamond structure from its 6 inputs to 6 outputs, takes the special form shown below:

$U=\left( \begin{matrix} \begin{matrix} * & * & * \\ * & * & * \\ * & * & * \end{matrix} & \begin{matrix} * & 0 & 0 \\ * & * & 0 \\ * & * & * \end{matrix} \\ \begin{matrix} * & * & * \\ 0 & * & * \\ 0 & 0 & * \end{matrix} & \begin{matrix} * & * & * \\ * & * & * \\ * & * & * \end{matrix} \end{matrix} \right)=\left( \begin{matrix} T & A \\ B & C \end{matrix} \right)$, (7)

where the black block is the desired 3x3 non-unitary matrix T, the green and the orange blocks correspond to a lower triangular matrix A and an upper triangular matrix B, respectively, and the blue block denotes matrix C.

To realize the transform matrix T on the diamond structure, we must construct a unitary matrix U as shown in (7). According to the unitary matrix definition,

$\begin{matrix} UU^{+}=\left( \begin{matrix} T & A \\ B & C \end{matrix} \right)\left( \begin{matrix} T^{+} & B^{+} \\ A^{+} & C^{+} \end{matrix} \right)=I, \\ U^{+}U=\left( \begin{matrix} T^{+} & B^{+} \\ A^{+} & C^{+} \end{matrix} \right)\left( \begin{matrix} T & A \\ B & C \end{matrix} \right)=I, \end{matrix}$ (8)

where $+$ is the conjugate transpose. Therefore, the following equations should be satisfied:

$\begin{matrix} TT^{+}+AA^{+}=I, \\ T^{+}T+B^{+}B = I, \\ BT^{+}+CA^{+}=0. \end{matrix}$ (9)

Because *A* is a lower triangular matrix, we can obtain *A* by decomposing $I-TT^{+}$ via the Cholesky decomposition. Similarly, *B* can be obtained by transposing the Cholesky decomposition of $I-T^{+}T.$ With the result of A and B, we can obtain C with

$C=-BT^{+}{{(A}^{+})}^{-1}.$ (10)

Now, to realize a non-unitary matrix *T*, we have constructed a unitary matrix *U* that satisfies the structural requirements of the diamond configuration, as shown in (7).

The final step is to determine all the $\theta$ and $\varphi$ for each MZI within the diamond mesh. This can be completed by applying the method proposed in Ref [3].

**Supplementary Information Note 5:** Advantage analysis for the diamond structure compared to alternative architectures.

The key challenge in implementing non-unitary matrices in MZI-cascaded meshes lies in realizing non-unitary operations using hardware that inherently provides unitary operations for multiple inputs. From the algorithm perspective, two methods are intuitive: (1) decomposing the non-unitary matrix into unitary matrices, and (2) expanding the non-unitary matrix into a unitary matrix. Method (1) refers to the widely used Singular Value Decomposition (SVD), and method (2) represents our proposed Lower-Upper Expansion (LUE), detailed in Supplementary Information Note 4. Structurally, MZI-cascaded meshes that can realize unitary operations for multiple inputs include the triangle, rectangle, and diamond configurations. Specifically, the unitary matrix provided by the diamond structure features lower and upper triangle matrices in its top-right and bottom-left sub-blocks, respectively. Consequently, there are five implementations for realizing non-unitary matrices in the MZI-cascaded mesh: SVD on triangle structure, SVD on rectangle structure, LUE on triangle structure, LUE on rectangle structure, and LUE on diamond structure.

Two merits are important: the footprint of the structure and the number of active MZIs in the structures (corresponding to energy consumption).

For fair comparison, we avoid using amplifiers or attenuators to implement diagonal matrices in the SVD methods. Instead, we implement all three matrices of the SVD in a single chip in which MZIs are implemented to realize the diagonal matrices and certain MZIs in Bar or Crossing states solely for light routing. Moreover, we compare these structures in their forward-only meshes, noting that their relative costs in recirculating meshes are similar.


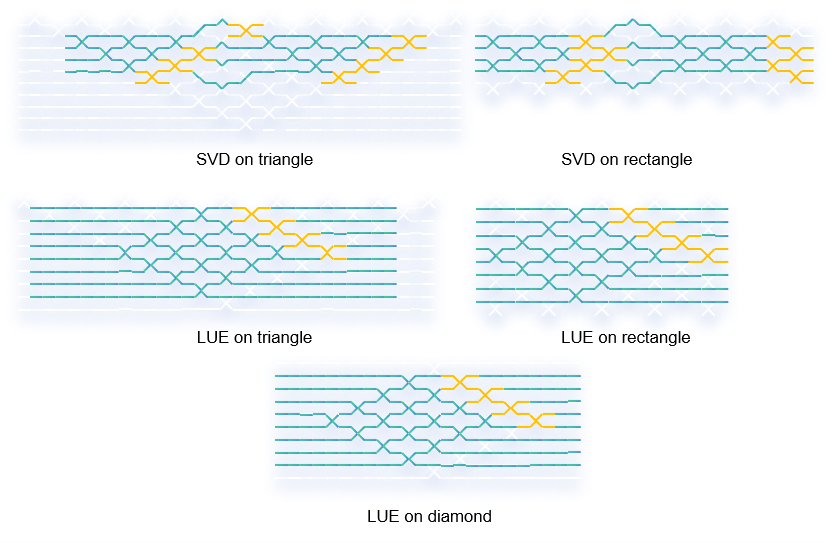


Figure S4 Implementations of non-unitary matrices realized in the unitary MZI-cascaded structures.

Figure S4 demonstrates examples of the five implementations for a 4×4 non-unitary. The results clearly show that the LUE on diamond structure achieves both the smallest footprint and the lowest number of MZIs. Generally, for an N×N non-unitary matrix, the footprints for the SVD on triangle structure, SVD on rectangle structure, LUE on triangle structure, LUE on rectangle structure, and LUE on diamond structure are $\left( 8N^{2}+14N+6 \right)LW,$ $\left( 6N^{2}+N-2 \right)LW$, $\left( 4N^{2}+4N+1 \right)LW$, $\left( 4N^{2}+4N+1 \right)LW$ and $\left( 2N^{2}+2N+1 \right)LW$, respectively, where $L$ and $W$ are the length and width of the MZI, respectively. And the numbers of active MZIs are $2N^{2}+5N+3$, $3N^{2}+\left\lceil N/2 \right\rceil-1$, $2N^{2}+3N+1$, $2N^{2}+2N+1$, and $N^{2}+2N+1,$respectively. The LUE on the diamond structure proves optimal. Additionally, when considering the diamond structure’s inherent triangle sub-block constraints and the more general applications of triangle and rectangle structures, LUE implementations on triangle and rectangle structures still demonstrate smaller footprints and fewer MZIs compared to their SVD counterparts.

**Supplementary Information Note 6:** Wavelength locking approaches: electronic vs. photonic solutions.

The wavelength locking of micro-ring modulators (MRMs) typically relies on feedback control systems that monitor MRM output signals and accordingly adjust the heating voltage. Here, we compare the conventional electronic approaches with our proposed photonic differentiator-based solution.

The conventional electronic approach employs CMOS circuits. Reference ^4^ presents a feedback-based wavelength locking technique, which monitors the optical modulation amplitude at the drop port of MRM via an integrated Ge PD, utilizes a specifically designed CMOS circuit to infer the wavelength deviation by the PD output slope changes, and adjusts the heater to control the MRM temperature for resonance wavelength aligning with the laser wavelength. The CMOS circuit employs tunable delay lines to compensate for the timing mismatch between the output of a bit counter after the modulation signal and the transimpedance amplifier (TIA) connected to the slope quantizer. Specifically, these tunable delays are implemented using digital delay blocks, providing a tuning range of up to 900 *ps,* whose working clock is competitive with the modulation signal baud rate. Therefore, such an approach requires an advanced process for high-speed digital circuits as well as high-power consumption. In this work, this approach achieves a power efficiency of $1.45 pJ {bit}^{-1}$. The demonstrated data transmission rate is $2 Gb s^{-1}$. Analog delay modules, which typically rely on resistive and capacitive components, tend to exhibit large delay times and are thereby unsuitable for wavelength locking of MRMs, where sub-nanosecond delays are required.

In contrast, the photonic differentiator-based approach doesn’t rely on advanced processes and high-speed digital circuits. We have realized the photonic differentiator-based approach in the proposed LightIN. It is fabricated using an 180 *nm* process, and the slope quantizer circuit (a host computer in the demonstrated experiments) can work in a direct current (DC) mode, enabling on-chip inter-symbol differential computation for data under baud rates from $5 Gb s^{-1}$ to $32 Gb s^{-1}$. The power consumption is estimated as $11 pJ {bit}^{-1}$ under the $32 Gb s^{-1}$ transmission, including power consumption to configure MZIs (three MZIs: $\pi$ phase shifts, two MZIs: $\pi/2$ phase shifts, 90 *mW* for $\pi$ phase shifts in current LightIN) and implement slope quantizer (0.1 *mW* as in the reference [3]). When we utilize the optimized MZI 2 *mW* to complete $\pi$ shifts (have been realized by our team) in the future LightIN, the energy efficiency will be significantly reduced to $0.28 pJ {bit}^{-1}$. Furthermore, the energy efficiency has the potential to achieve below $10 fJ {bit}^{-1}$, when the photonic differentiator is application-specifically designed for a fixed data rate. Only the power supplied to the slope quantizer is required without the need for photonic differentiators, except for a very low power to calibrate delay variations from fabrication.

We compare the performance of these approaches in the following table. Although a more comprehensive review of the state-of-the-art could be beneficial, the current analysis sufficiently demonstrates the advantages of the photonic differentiator-based approaches proposed in our work.

Table SI 1 Comparison of different delay modules for wavelength locking of MRMs

|  | Analog delay module scheme | Digital delay line scheme [3] | This work and its potential future implementations |
| --- | --- | --- | --- |
| Delay range | > 1 *us*,  cannot be applied in wavelength locking of MRM | 1. 900 *ps* 2. ~50 *ps* under specific design for a fixed data rate | 1. Programmable as required in LightIN 2. Application-specific designed as low as 10^-3^ *ps* for a fixed data rate |
| Power | \ | 1. $1.45 pJ {bit}^{-1}$ in measurement 2. ~$200 fJ {bit}^{-1}$ under application specific design for a fixed data rate | 1. $11.25 pJ {bit}^{-1}$ with current LightIN 2. $0.28 pJ{bit}^{-1}$ with future LightIN constructed by optimized MZIs 3. ~$10 fJ {bit}^{-1}$ with application-specific photonic differentiator for fixed data rate |
| Data rate | \ | $2 Gb s^{-1}$ | $5 Gb s^{-1}\sim32 Gb s^{-1}$ in the demonstration, potentially higher in future LightIN and application-specific photonic differentiators |
| Process | \ | 40 *nm* CMOS | 1. *nm* |

**Supplementary Information Note 7:** Path-independent loss (PILOSS) non-blocking switch in the MZI-based recirculating square mesh.

Although the current MZI-based recirculating square mesh cannot demonstrate the PILOSS switch due to its limited mesh size, it has the potential to realize such a switch in principle. The following figure illustrates a topology example for the 6-input-6-output switch implemented in the MZI-based recirculating square mesh.


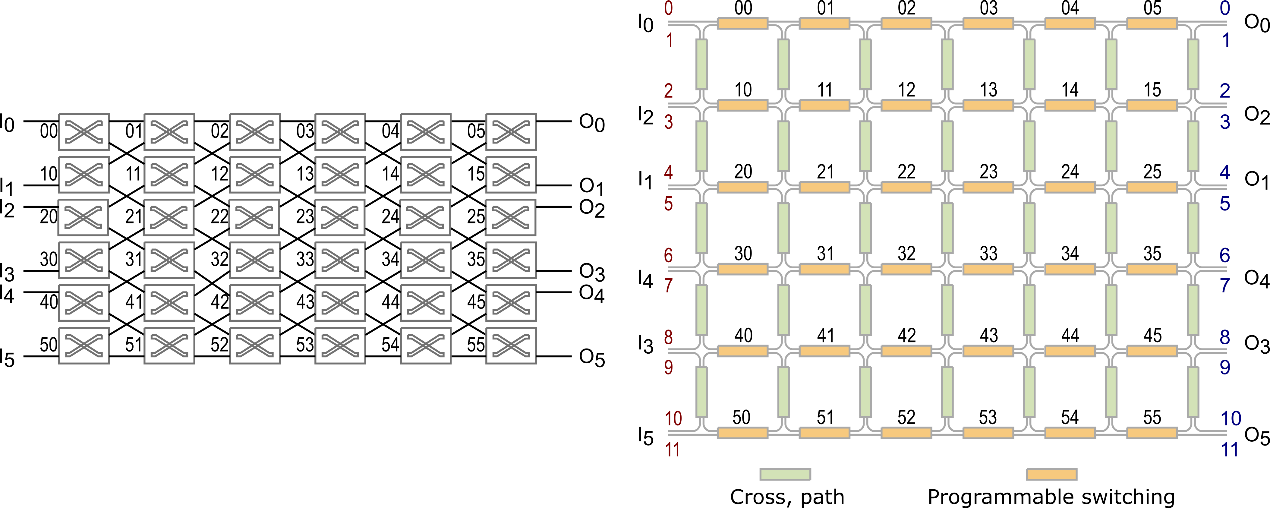


Figure S5 The PILOSS non-blocking switch structure and its topology in the MZI-based recirculating square mesh.

**Supplementary Information Note 8:** Measurement and analysis for the initial phase shift distribution in the MZI-based square meshes.

To set the phase shifter variations for the photonic physical unclonable functions in the Lumerical-based simulation platform, we have measured the initial states of MZIs from two dies using the proposed TCA framework at room temperature (25 *°C*). The measured phase shift initial states are presented in Figure S6. The fitting results confirm a Gaussian Distribution. The mean ($\mu$) and standard deviation ($\sigma$) values are -0.8457 *rad* and 1.1815 *rad*, respectively. These statistical parameters were then used to generate random initial states for MZIs in our simulation platform to evaluate the PUF performance.


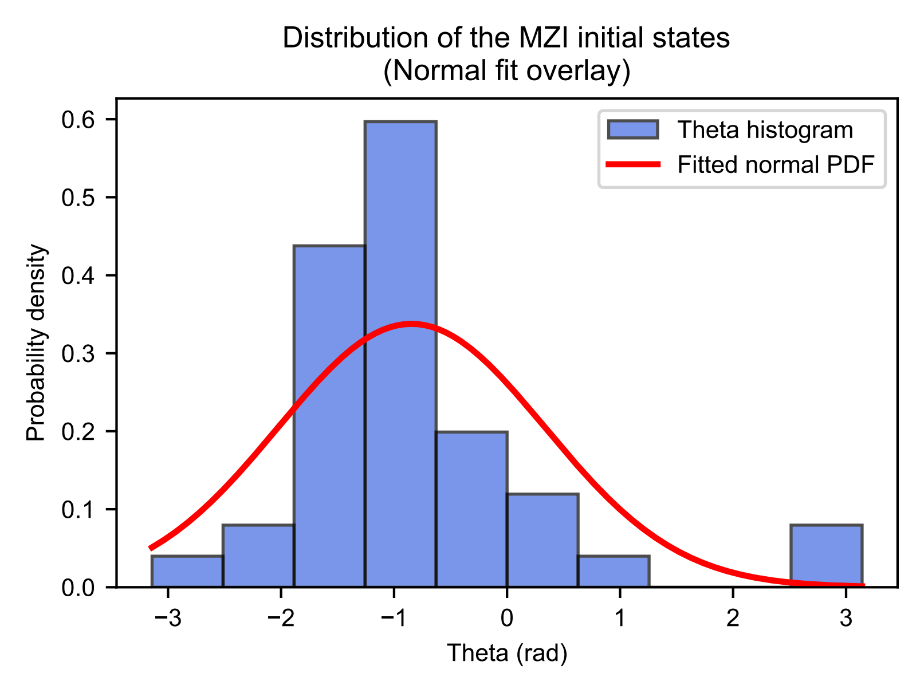


Figure S6 The distribution of the MZI initial phase states.

**Supplementary Information Note 9:** A quantitative analysis for the potential scale of the MZI-based square mesh.

While the current LightIN has a size of 4×4, it has the potential to scale to a larger size. The size is primarily constrained by the output signal quality, which determines the performance of the applications implemented on the LightIN.


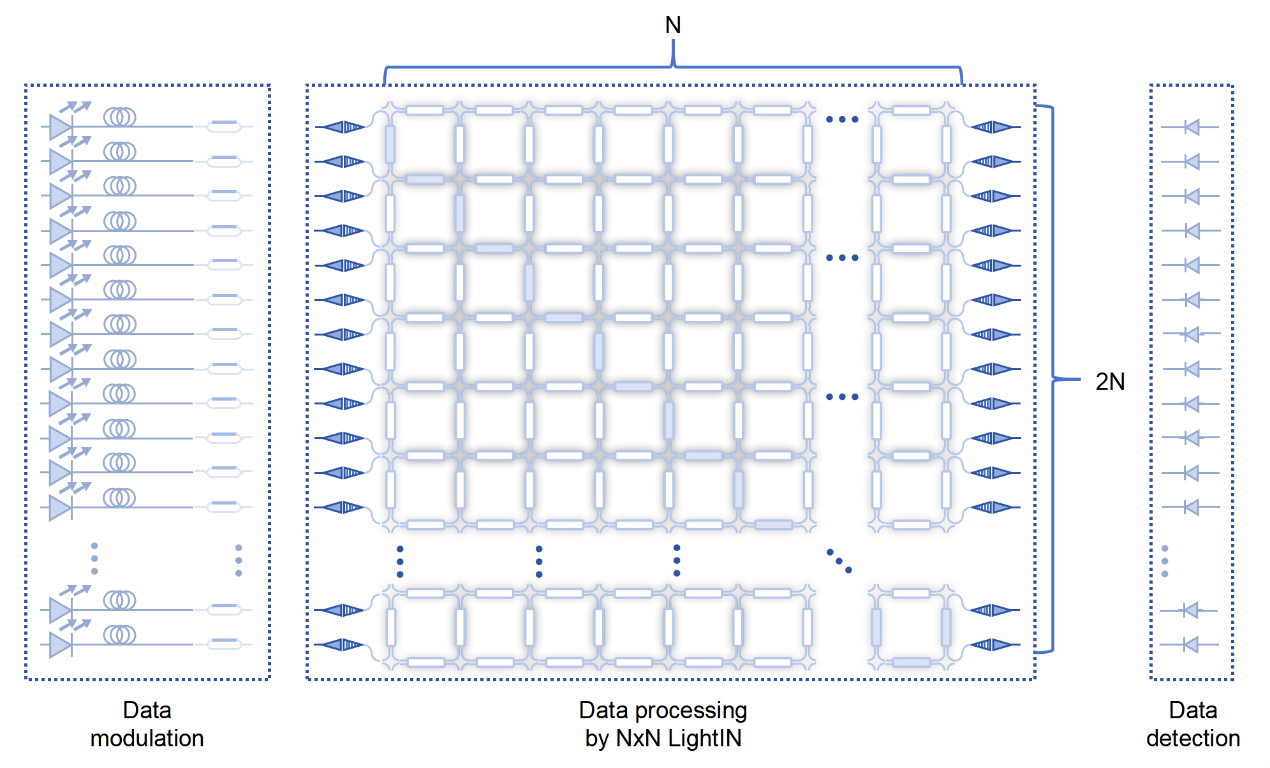


Figure S7 The diagram of the on-chip structure of the N×N LightIN, where the longest path for computing operation is indicated by the blue-highlighted MZIs.

Figure S7 presents the analyzed structure of an N×N MZI-based square mesh in the following, which consists of the edge couplers, MZIs, and bending waveguides. Take the computing operation as an example, in which light travels through one of the longest paths, consisting of a pair of edge couplers, $2N+1$ MZIs, and $2N+2$ bending waveguides, and experiences one of the largest attenuations. The input and output edge couplers introduce a loss of 2${IL}_{EC}$. The MZI insertion loss ${IL}_{MZI}$ is primarily determined by waveguide and phase shifter losses, ${IL}_{PS}$, and the insertion losses, ${IL}_{ADC}$, of two adiabatic directional couplers (ADCs). The bending waveguide insertion loss is ${\frac{\pi}{2}\eta}_{wg}R_{s}$, where $R_{s}$ is the radius of the bending waveguide and $\eta_{wg}$ represents the optical intensity attenuation in the Si waveguide. In addition to the on-chip impacts, the fibers that connect the modulators to the LightIN chip and the LightIN chip to the PDs introduce an attenuation of ${2Att}_{fiber}$. Therefore, when the modulated signal has a power of $P_{Mod}$, the optical intensity received by PD is

$$\begin{aligned} P_{PD}=P_{Mod}-{Att}_{fiber}-{IL}_{EC}-\left( 2N+1 \right){IL}_{MZI}-\left( N+1 \right)\eta_{wg}\pi R_{s}-{IL}_{EC}-{Att}_{fiber.}\#\left( 11 \right) \end{aligned}$$

It can be converted to n-bit signals, which follows the equation as^5^:

$$\begin{aligned} n=\frac{1}{6.02}\left[ 20\log_{10} \left( \frac{RP_{PD}}{\left[ \sqrt{2q\left( RP_{PD}+I_{d} \right)+\frac{4kT}{R_{L}}+R^{2}P_{PD}^{2}RIN}+\sqrt{2qI_{d}+\frac{4kT}{R_{L}}} \right]\sqrt{\frac{DR}{\sqrt{2}}}} \right)-1.76 \right]\#,\left( 12 \right) \end{aligned}$$

where the parameters are defined and set as in the Table SI 2.

Based on the derivation, when the insertion losses achieve the values shown in Table SI 2, which are obtained from the available Process Design Kit, a size of 32×32 MZI-based square mesh can complete matrix multiplications with a resolution of 4 bits. Furthermore, it has the potential to scale to 64×64 with a bit resolution exceeding 5 bits when the ADC insertion loss reaches 0.01 dB (which is theoretically possible and has been realized in simulation).

Table SI 2 The summary of the parameters in this note

| Parameter | Description | Value |
| --- | --- | --- |
| $R$ | PD responsivity | $1.2 A W^{-1}$Ref$[ ADDIN ZOTERO\_ITEM CSL\_CITATION \{"citationID":"bfVWIGG2","properties":\{"formattedCitation":"\backslash\backslash super 6\backslash\backslash nosupersub\{\}","plainCitation":"6","noteIndex":0\},"citationItems":[\{"id":12704,"uris":["http://zotero.org/users/7145154/items/8IVL9XJQ"],"itemData":\{"id":12704,"type":"paper-conference","container-title":"2020 European Conference on Integrated Optics","title":"Silicon-germanium pin photodiodes with double heterojunction: High-speed operation at 10 Gbps and beyond","author":[\{"family":"Benedikovic","given":"Daniel"\},\{"family":"Virot","given":"Léopold"\},\{"family":"Aubin","given":"Guy"\},\{"family":"Hartmann","given":"Jean-Michel"\},\{"family":"Amar","given":"Farah"\},\{"family":"Szelag","given":"Bertrand"\},\{"family":"Le Roux","given":"Xavier"\},\{"family":"Alonso-Ramos","given":"Carlos"\},\{"family":"Crozat","given":"Paul"\},\{"family":"Cassan","given":"Eric"\},\{"literal":"others"\}],"issued":\{"date-parts":[["2020"]]\}\}\}],"schema":"https://github.com/citation-style-language/schema/raw/master/csl-citation.json"\}$6$]$ |
| $I_{d}$ | Dark current | $35 nA$ ^7^ |
| $k$ | Boltzmann constant | $1.380649\times{10}^{-23} J K^{-1}$ |
| $T$ | Absolute temperature | $300 K$ |
| $R_{L}$ | Load resistance | $50 \Omega$ |
| $RIN$ | Relative intensity noise | $-140 dB {Hz}^{-1}$ |
| $q$ | Electric quantity | $1.6\times{10}^{-16} C$ |
| $DR$ | Data rate | $1 GB s^{-1}$ |
| $P_{Mod}$ | Optical power after modulation | $10 dBm$ |
| ${Att}_{fiber}$ | Fiber attenuation | $\sim0 dB (0.18 dB {km}^{-1})$ |
| ${IL}_{EC}$ | Insertion loss from edge couplers | $1.3 dB$ ^*^ |
| ${IL}_{MZI}$ | The insertion loss of one MZI | $0.25 dB$  $(0.1 dB$per ADC^8^, $200 \mu m$per arm^*^) |
| $\eta_{wg}$ | The optical intensity attenuation in the Si waveguide | $2.3 dB {cm}^{-1}$ ^*^ |
| $R_{s}$ | The radius of bending waveguides | $30 \mu m$^*^ |

^*^ Values from the measurement results of the up-to-date PDK of NOEIC (our affiliation).

**Supplementary Information Note 10:** The modification and expansion of the square mesh core to realize multi-core large-scale integration.

To enhance the system size, besides increasing the size of one chip, we can scale the whole system by interconnecting them in one package through the lens coupling package. Figure S8 demonstrates the modification for the MZI-based square mesh core and its multi-core scaling approach. Through this scheme, under the condition of acceptable coupling and insertion losses, we could construct a large-scale P-FPGA system with multiple photonic cores.


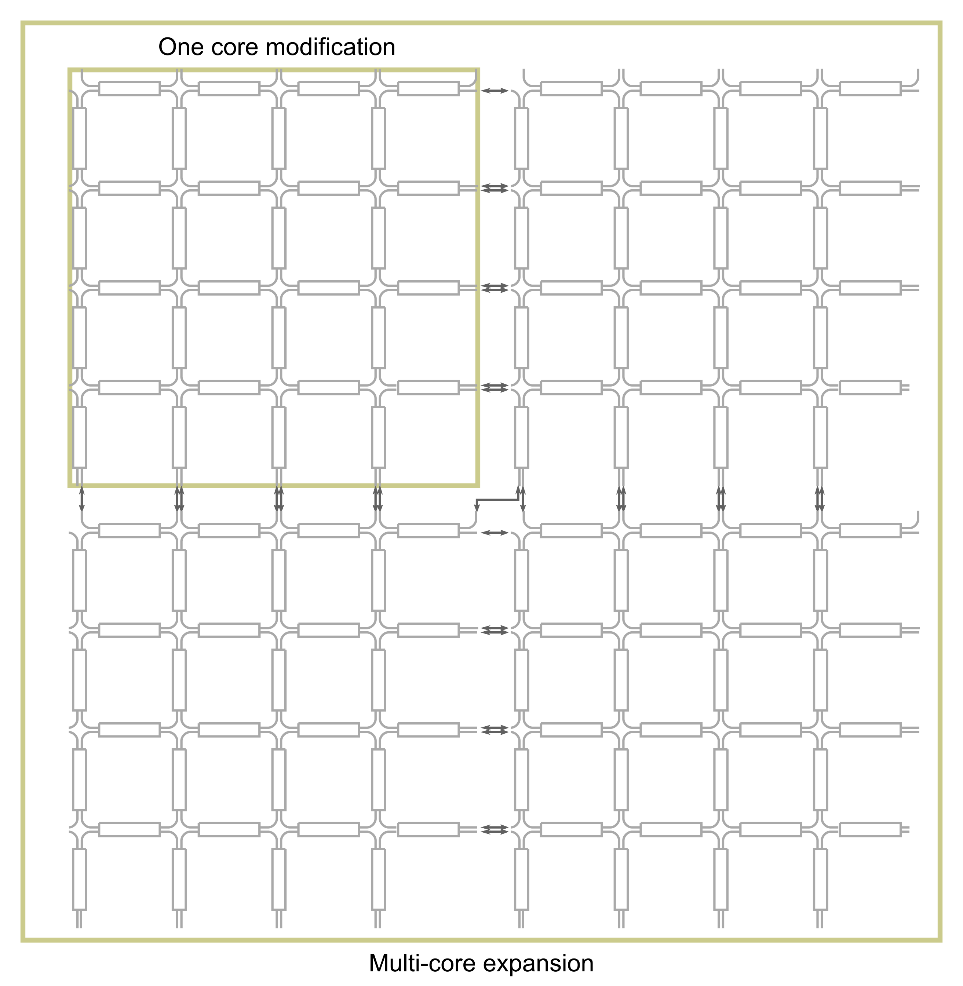


Figure S8 The conceptual diagram of modification and expansion for the MZI-based squared mesh core.

**Supplementary Information Note 11:** A possible topology for LightIN to simultaneously lock wavelengths of four micro-ring modulators.

In the scenario of Optical I/O, multiple micro-ring modulators (MRMs) work simultaneously. Therefore, it is required to construct multiple differentiators in the MZI-based recirculating square mesh to individually lock the wavelengths for the multiple MRMs. In the following figure, we demonstrate a possible topology for four differentiators.


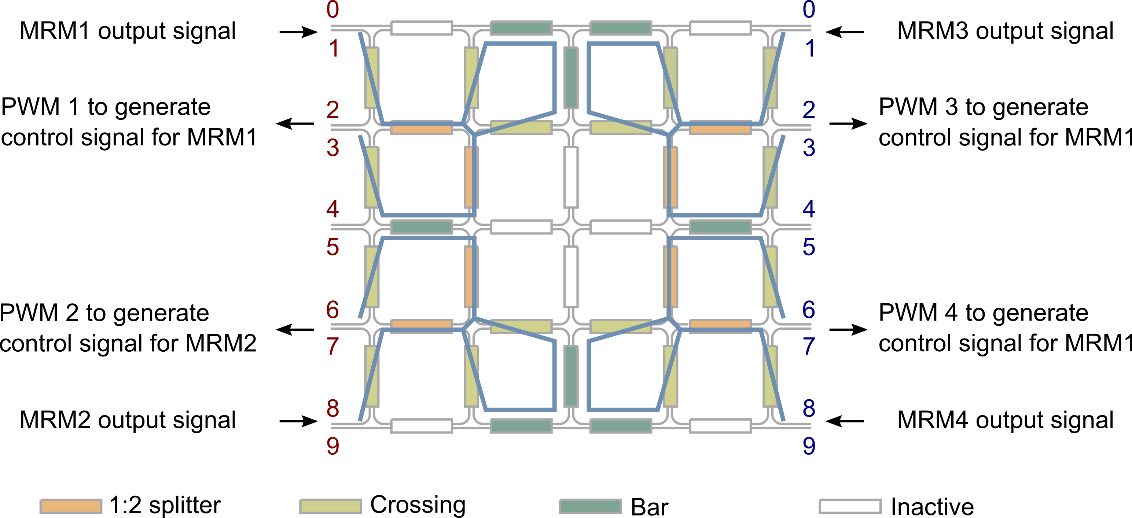


Figure S9 A possible topology implemented in the LightIN for wavelength locking of four micro-ring modulators.

Reference

1. Hamerly, R., Basani, J. R., Sludds, A., Vadlamani, S. K. & Englund, D. Towards the information-theoretic limit of programmable photonics. *arXiv preprint arXiv:2408.09673* (2024).

2. Hua, S. *et al.* An integrated large-scale photonic accelerator with ultralow latency. *Nature* **640**, 361–367 (2025).

3. Reck, M., Zeilinger, A., Bernstein, H. J. & Bertani, P. Experimental realization of any discrete unitary operator. *Phys. Rev. Lett.* **73**, 58–61 (1994).

4. Agarwal, S. *et al.* Wavelength locking of a Si ring modulator using an integrated drop-port OMA monitoring circuit. *IEEE Journal of Solid-State Circuits* **51**, 2328–2344 (2016).

5. Al-Qadasi, M., Chrostowski, L., Shastri, B. & Shekhar, S. Scaling up silicon photonic-based accelerators: Challenges and opportunities. *APL Photonics* **7**, (2022).

6. Benedikovic, D. *et al.* Silicon-germanium pin photodiodes with double heterojunction: High-speed operation at 10 Gbps and beyond. in *2020 European Conference on Integrated Optics* (2020).

7. Giewont, K. *et al.* 300-mm monolithic silicon photonics foundry technology. *IEEE Journal of Selected Topics in Quantum Electronics* **25**, 1–11 (2019).

8. Chrostowski, L. *et al.* Silicon photonic circuit design using rapid prototyping foundry process design kits. *IEEE Journal of Selected Topics in Quantum Electronics* **25**, 1–26 (2019).
